# Five Hypotheses on the Origins of Temperature Dependence of ^77^Se NMR Shifts in Diselenides

**DOI:** 10.1021/acs.inorgchem.4c01025

**Published:** 2024-06-14

**Authors:** Alexandra C. Koziel, Marco Bortoli, Matthew Tremblay, Yilun Zhao, Laura Orian, Zhongyue J. Yang, Nathan D. Schley, Janet E. Macdonald

**Affiliations:** † Department of Chemistry, Vanderbilt University, 1234 Stevenson Center Lane, Nashville, Tennessee 37240, United States; ‡ Dipartimento di Scienze Chimiche, Università degli Studi di Padova, Via Marzolo 1, 35131 Padova, Italy

## Abstract

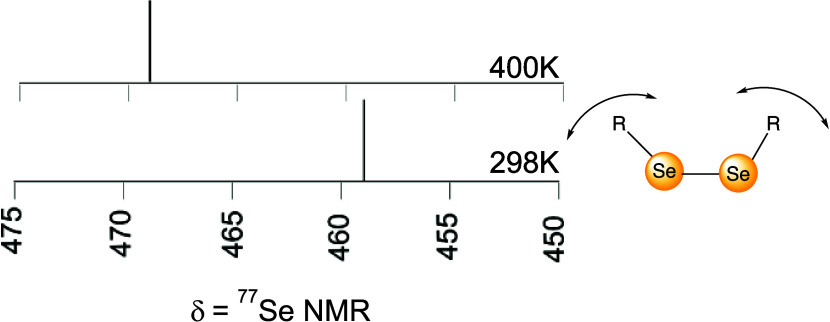

Notable thermal shifts in diselenides have been documented in ^77^Se NMR for more than 50 years, but no satisfactory explanation has been found. Here, five hypotheses are considered as possible explanations for the large temperature dependence of the ^77^Se chemical shifts of diaryl and dialkyl diselenides compared to monoselenides and selenols. Density functional theory calculations are provided to bolster hypotheses and better understand the effects of barrier height and dipole energies. It is proposed that the temperature dependence of diselenide ^77^Se NMR chemical shifts is due to rotation around the Se–Se bond and sampling of twisted conformers at higher temperatures. The molecular twisting is solvent dependent; here, DMSO-*d*_6_ and toluene-*d*_8_ were evaluated. No correlation was established between *para*-substituents on diaryl diselenides and the magnitude of the change in the ^77^Se NMR shift (Δδ) with temperature.

## Abstract 2

Five hypotheses were explored to find an answer for why diaryl and dialkyl diselenides shift more than monoselenides and selenols when heated *in situ*^77^Se NMR. Density functional theory calculations are provided to strengthen these hypotheses and better understand the effects of barrier height and dipole energies. It is proposed that diselenides shift *in situ*^77^Se NMR due to rotations around the Se−Se bonds and the sampling of twisted conformers at higher temperatures.

## Introduction

Selenium is most commonly studied for its role in human body functions and proteins,^1^ however, there are numerous organic and inorganic chemistry methods that depend on the chemistry of selenium.^2,3^ For example, some selenium-containing molecules are thermochromic, and recently the origin of this property in both diphenyl diselenide and ditelluride was studied determining both inter- and intramolecular factors playing a role.^4^

Characterization of organoselenium compounds is facilitated by ^77^Se nuclear magnetic resonance (NMR).^5−9^ Selenium has six naturally occurring isotopes, but selenium-77 is the only NMR active nucleus being spin 1/2 and 7.63% abundant.^10^^77^Se has a wide spectral range of approximately 3000 ppm and is sensitive to subtle changes in the electronic structure of a molecule. Gaining a further understanding of ^77^Se NMR spectroscopy will offer new opportunities to study organic, inorganic, and biological structures and functions.

For *para-*substituted diaryl diselenides, it has long been established that there is a strong correlation between the Hammett parameter and the ^77^Se NMR chemical shift. Previously, studies of substituted diselenides have been performed, and strong room temperature correlations have been established.^8,9^ Electron-withdrawing groups cause a downfield chemical shift. In this manner, the chemical shift can be a measure of electron density on selenium. More recently, Sørensen et al. showed a similar phenomenon in selenocarbamates with ^1^H, ^13^C, and ^77^Se NMR.^6^ They also observed a strong linear correlation between the *para* Hammett parameters that describe the electron-withdrawing effect of the substituent and the chemical shift of each molecule showing a more universal trend in ^77^Se NMR.^6^

Curiously, about 50 years ago, Lardon et al. noted that diaryl diselenides had large thermal chemical shifts compared to that of selenols or diaryl selenides. Oliver et al. also studied thermal shifts in bulky diselenides. They studied rotation about the Se–Se and Se–C bonds and proposed two rotational processes that overcome a 15 kcal/mol rotational barrier.^11^ Eggert et al. noted similar behavior in dialkyl diselenides. Lardon did not attempt an explanation, and Eggert noted additional line broadening but explained it as thermal gradients in the NMR tube. The mystery has been left unsolved.

However, given the importance of the diselenide functional group in biology, drug delivery, and synthetic organic transformations, it is important to understand the source of the thermal shifts of diselenides in ^77^Se NMR, as it may give clues to temperature-dependent behavior in these arenas. Stimuli-responsive Se–Se bonds in polymers are studied for cancer drug delivery applications.^12^ Tumors tend to have a redox microenvironment that can either reduce diselenides to selenol (RSeH) with high concentrations of glutathione or oxidize them to selenate (SeO_4_^2–^) with reactive oxygen species in the hypoxic environment of the tumor. Selenocystine, an essential amino acid, exists as a diselenide^12^ before incorporation into selenoproteins in all three branches of life: eukarya, archaea, eubacteria, and even some viruses.^13^ Diphenyl diselenide has been used as a catalyst for numerous transformations including in the production of isocoumarins, which are a scaffold for many natural products.^14^ The temperature dependence of the ^77^Se NMR is another handle beyond the chemical shift to understand the structural dynamics of diselenide-containing molecules.

Here, we examine a collection of nine diselenides and study their temperature-dependent ^77^Se NMR chemical shifts in various solvents. In almost all cases, a downfield shift occurs on heating, but in one case, a positive shift was observed. Several hypotheses for the thermal shift are presented, including solute–solute interactions, fast equilibrium with a second isomer, electronic effects, solvent interactions, and conformational rotation. A combination of experiment and theory is used as evidence against or in support of each hypothesis, with the preponderance of evidence supporting conformational rotation around the Se–Se bond and enhanced sampling of higher energy twisted conformers with elevated temperature as the most likely explanation.

## Experimental and Theoretical Methods

### Materials

All materials were used as received without further purification unless otherwise noted. 1,4-iodotoluene, 1,4-iodoanisole, 1,4-iodoaniline, 1,4-iodobromobenzene, 1,4-iodofluorobenzene, diphenyl diselenide, iodine, selenium, potassium hydroxide, copper oxide nanopowder <50 nm, anhydrous tetrahydrofuran, ACS grade hydrochloric acid, anhydrous dimethyl sulfoxide, ethyl acetate, sodium sulfate, sodium borohydride, tetrahydrofuran (THF), bromodecane, magnesium turnings, sodium selenide, dimethylformamide, anhydrous ethanol, dimethylformamide, α,α′-dibromo-*o*-xylene, Meerwein’s salt ([Me_3_O]BF_4_), dichloromethane, acetonitrile, dioctyl ether, ether and deuterated chloroform (Sigma-Aldrich), deuterated dimethyl sulfoxide, deuterated toluene, dibenzyl diselenide (TCI Chemicals), potassium hydroxide and diethyl ether (Fisher Scientific) 1-chloro-4-iodobenzene, 1-iodo-2-methylbenzene, and 2-bromo-1,3,5-triisopropylbenzene (Oakwood Chemicals).

All syntheses were performed on a Schlenk line under nitrogen. A heating mantle setup with a proportional integrative derivative controller (PID) and a thermocouple were used for all syntheses. All known compounds were confirmed by comparison to their literature ^1^H NMR. *Hazards include the fact that diselenides are stench compounds. Proper precautions, including using a well-ventilated hood and cleaning all glassware thoroughly with dilute bleach, should be taken. No other uncommon hazards are noted.*

### Synthesis of Selenirenium Salts

Using a reported procedure,^15^ Ph_2_Se_2_ or Bn_2_Se_2_ (10 mmol) was treated with Me_3_O^+^BF_4_^–^ (10 mmol) in 5 mL CH_2_Cl_2_. The mixture was stirred for 2 h in a nitrogen-filled glovebox (temperature in the glovebox was ca. 32 °C). The selenium salt was precipitated by the dropwise addition of 50 mL of dry ether. The solid was filtered, washed with ether, and the resulting product was taken up in 4 mL CH_3_CN and recrystallized through the addition of 50 mL dry ether at −10 °C. The resulting crystal structures are shown (Figure S1).

### General Synthesis of Substituted Diselenides

A reported procedure from Braga et al.^16^ was followed for the synthesis of *para-*substituted diselenides.

In a 50 mL three-neck flask, 2.0 mmol of aryl iodide, 4.0 mmol of selenium, 4.0 mmol KOH, and 0.2 mmol CuO nanopowder <50 nm were combined. The flask was placed under vacuum at room temperature for 30 s before refilling with nitrogen three times. 4.0 mL of anhydrous DMSO was then injected, and the flask was heated to 90 °C while stirring. The reaction was monitored by thin layer chromatography (30% ethyl acetate: 70% hexane for all products except 2-bis (4-chlorophenyl)diselenide, which was run with 100% petroleum ether). The reaction times varied between 2–4 h. The reaction was removed from heat and allowed to cool before it was mixed with deionized water in a separation funnel. Three extractions with ethyl acetate were then performed. The organic portions were combined and washed three times with brine. The organic layer was dried with sodium sulfate. The solution was then filtered into a pear-shaped flask and the solvent removed under reduced pressure. When necessary, a silica gel column was performed with the solvent systems listed above. The products were confirmed by comparison of their ^1^H NMR to existing reports. 1,2-bis(4-bromophenyl)diselenide (yellow powder, yield 81%), 1,2-bis(4-methoxyphenyl)diselenide (orange crystals, yield 75%), 1,2-bis(4-fluorophenyl)diselenide (rust-colored oil, yield 83%), 1,2-di-*p*-tolyldiselenide (orange crystals, yield 85%), 1,2-bis (4-chlorophenyl)diselenide (orange oil, yield 77%), and 1,2-di-*o*-tolyldiselenide (orange oil, yield 87%).

### Synthesis of Didodecyl Diselenide (DDSe_2_)

The synthesis of didodecyl diselenide was adapted from a previously published method.^17^ Briefly, selenium powder (0.117 mol, 9.3 g) and NaBH_4_ (0.259 mol, 9.8 g) were mixed to form Na_2_Se_2_, which was then reacted with bromododecane (0.265 mol, 58.6 g, 56.5 mL) in THF (280 mL) at 50 °C for 18 h to form the diselenide. The product was collected through extraction with dichloromethane and brine and dried by using a rotary evaporator. The crude product was then dissolved in heptane and then crystallized by adding ice-cold isopropyl alcohol. The final product (25.9 g, 40%) was dried under a vacuum prior to use. The product was stable and stored in ambient conditions.

### Synthesis of Didodecyl Selenol (DDSeH)

This synthesis was performed according to a previously published method.^17^ Anhydrous ethanol (0.36 mol, 16.6 g) was added to a mixture of NaBH_4_ (0.119 mol, 4.5 g) and elemental selenium powder (0.059 mol, 4.7 g). After a white-gray solid was formed, anhydrous dimethylformamide (100 mL) was added dropwise. Formic acid (0.122 mol, 5.6 g) was then added, followed by bromododecane (0.057 mol, 12.5 g). After an overnight reaction, the mixture was hydrolyzed with 10% HCl and extracted with ether. Pure dodecyl selenol was obtained by distillation (boiling point 120 °C). The product was stored under N_2_ in a glovebox to prevent oxidation.

### Synthesis of Bis((2,4,6-triisopropyl)phenyl) diselenide

A reported synthesis from Zhang et al.^18^ was followed by adding magnesium turnings (0.72 g, 30 mmol) and a trace amount of iodine to a dry round-bottom flask under a nitrogen atmosphere. To this, 50 mL of dry tetrahydrofuran was added. After, 2-bromo-1,3,5-triisopropylbenzene was injected (7.0 mL, 30 mmol). This suspension was stirred in an oil bath at 50 °C for 2 h. Selenium powder (2.37 g, 30 mmol) was then added, and the reaction was heated at 50 °C and stirred overnight. The reaction was quenched with 50 mL of 0.1 M HCl and then extracted with diethyl ether (3 × 50 mL). The organic fraction was dried with magnesium sulfate and dried *in vacuo* yielding an orange oil. The orange crystalline product was yielded from a hot ethanol recrystallization. (Yield 31%)

### Synthesis of 1,4-Dihydrobenzo[*d*][1,2]diselenine

A procedure was adapted from Lim et al.^19^ to give a cyclic diselenide. A mixture of Se powder (79 mg, 1.0 mmol) and ground NaOH (40 mg, 1.0 mmol) was stirred in dimethylformamide (1.5 mL). NH_2_NH_2_·H_2_O (36 mL, 0.75 mmol) was then added under N_2_. This mixture was stirred for 2 h at 25 °C. During this time, the mixture turned a brown, red color. At this point, a solution of α,α′-dibromo-o-xylene (158 mg, 0.6 mmol) in 33 mL of dimethylformamide was added. The reaction proceeded for an additional 2 h at 25 °C. Deionized water (30 mL) was then added to the reaction, and extractions were performed using CH_2_Cl_2_ (3 × 30 mL). The resulting organic product was washed with brine (30 mL) and dried with MgSO_4_. Finally, the product was concentrated *in vacuo*, resulting in a yellow oil. (Yield 64%)

### Characterization

All NMR spectra, unless otherwise noted, were collected on a 500 MHz Bruker console equipped with a 11.7 T Oxford magnet and a 5 mm Z-gradient broadband (BBFO) probe. The samples were diluted with 600 μL of a deuterated solvent. When noted, 0.05 mmol of Ph_2_Se was added as an internal standard. All spectra were recorded at room temperature, unless otherwise noted. Heated and cooled NMR studies were performed in DMSO-*d*_6_ or toluene-*d*_8_. DMSO-*d*_6_ was chosen as it was readily available; signs of oxidation in the ^77^Se NMR were not seen. Temperatures ranged from 298 to 400 K for heated experiments and 253 to 298 K for the cooled experiments.

### Ultraviolet–Visible Spectroscopy

Diphenyl and dibenzyl diselenide were dissolved in toluene in separate quartz cuvettes, and room-temperature spectra of both diphenyl and dibenzyl diselenide were obtained. The diselenides in quartz cuvettes were then cooled in salted ice baths for 30 min. Another UV–vis spectrum was taken. Finally, the samples were heated at 100 °C for 30 min, and a final UV–vis spectrum was taken (Figure S3).

### In Situ Infrared Spectroscopy of Diaryl Diselenides

Using a ReactIR IC10 *in situ* IR experiments were run. 0.65 mmol of a diselenide in 30 mL of dioctyl ether was loaded into a two-neck flask in the glovebox. The instrument’s mirrors were aligned, and a background scan was taken. The reaction vessel was heated from room temperature to 160 °C in an oil bath, and IR scans were taken every minute (3800–800 cm^–1^, Figure S2).

### Theoretical Methods

DFT calculations were carried out with the Gaussian 16 software.^20^ All atoms have been described using Dunning’s correlation consistent basis set of triple ς quality (cc-pVTZ). This level of theory is here denoted as B3LYP/cc-pVTZ.^21−24^ To take into account solvation effects, the SMD solvation model^25,26^ is employed to mimic toluene and DMSO (level of theory SMD-B3LYP/cc-pVTZ). Frequency analysis showed that all fully optimized minima reported had only real frequencies. Constrained geometry optimization restraining the C–Se–Se-C dihedral to either 0° or 180° was employed to explore the rotation around the Se–Se bond. Constrained scans where only the relevant phenyl ring dihedral was free to rotate were started from the fully optimized minimum geometry for each substituted diphenyl diselenide and carried out for a total of 360° in 10° steps at the B3LYP/cc-pVTZ level of theory. Transition structures corresponding to the maxima of the obtained energy profiles were extracted for analysis. Tables S2 and S3 show the coordinates from these calculations.

## Results and Discussion

**Figure 1:**
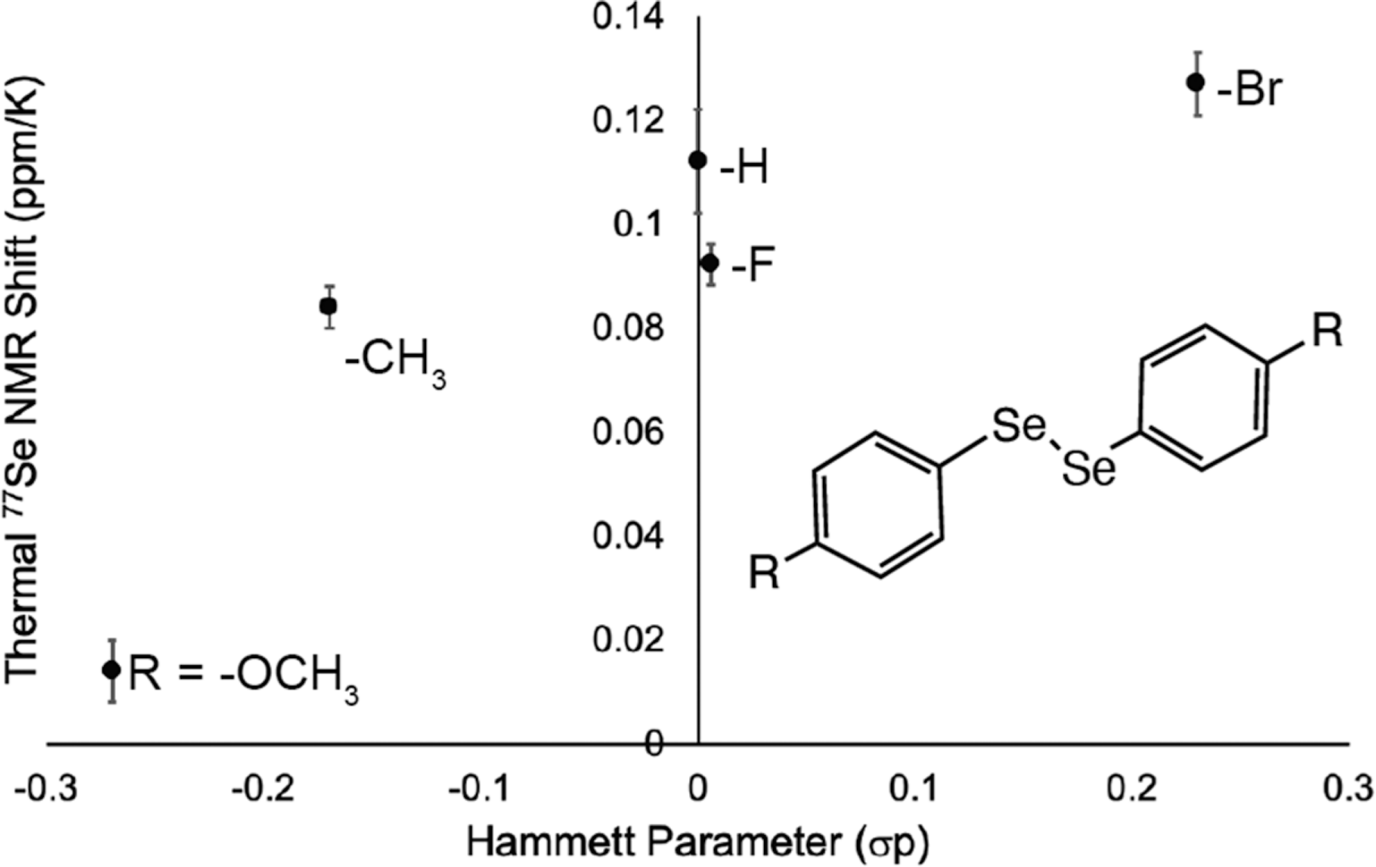
Thermal ^77^Se NMR shift of several *p-*substituted aryl selenides versus Hammett parameter of *para-*substituted diselenides in DMSO-*d*_6_.

**Figure 2:**
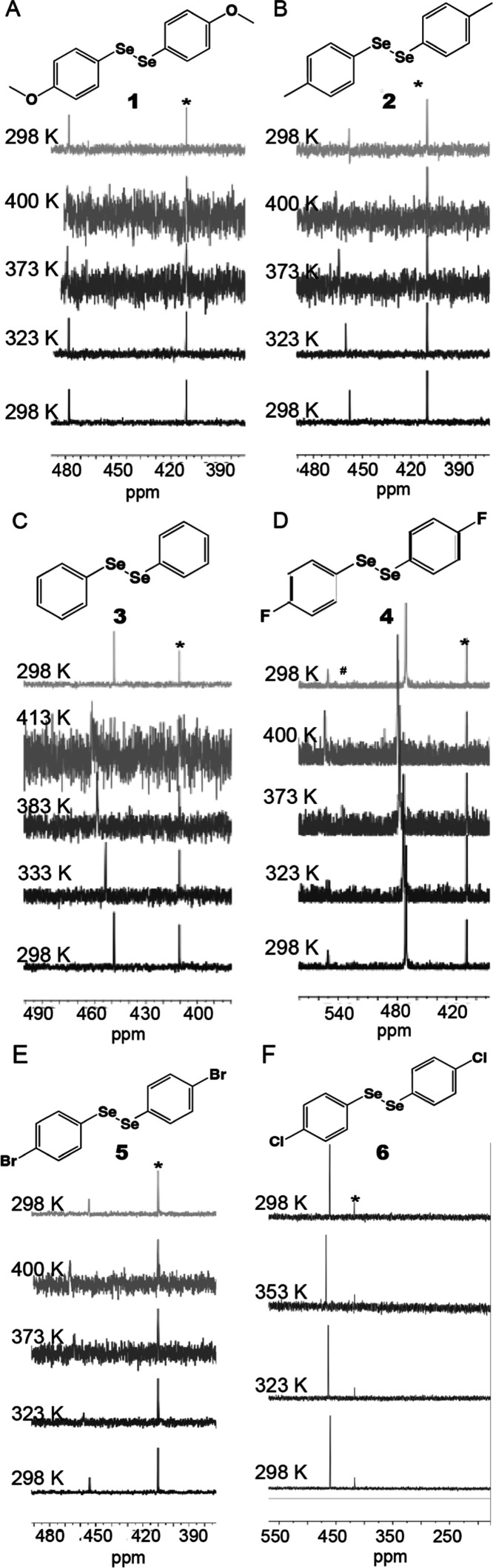
Heated ^77^Se NMR with Ph_2_Se internal standard (marked with *) of (A) 1,2-bis(4-methoxyphenyl)diselenide, (B) 1,2-di-*p*-tolyldiselenenide, (C) diphenyl diselenide, and (D) 1,2-bis(4-fluorophenyl)diselenide. As marked with #, a monoselenide byproduct formed. Minimal shifting was seen in the monoselenide peak. (E) 1,2-bis(4-bromophenyl)diselenide and (F) 1,2-bis(4-chlorophenyl)diselenide (in toluene-*d*_8_). All NMR preparations were performed in DMSO-*d*_6_ unless otherwise noted. Full ^77^Se and ^1^H NMR spectra are available in Figures S5 and S6. Figure S9 plots temperature-dependent chemical shifts in DMSO-*d*_6_ and Figure S10 plots temperature-dependent chemical shifts in toluene-*d*_8_.

A collection of *para-*disubstituted aryl diselenides all exhibited linear downfield shifts in ^77^Se NMR with increasing temperatures between 298 to 400 K relative to the diphenyl selenide internal standard (410 ppm, DMSO-*d*_6_) (Figures 1 and 2 and Table 1). The bromine-substituted diselenide shifted the most, with a change in chemical shift of 0.104 ppm/K. The anisole-substituted diselenide shifted the least, 0.066 ppm/K. The order of shifts from largest downfield shift to smallest was -Br > -H > −CH_3_ > -Cl > -*F* > -OCH_3_. All chemical shifts returned to their original position when the temperature returned to 298 K, which indicates that if a chemical transformation is involved, it is a thermally driven equilibrium.

**Table 1:**
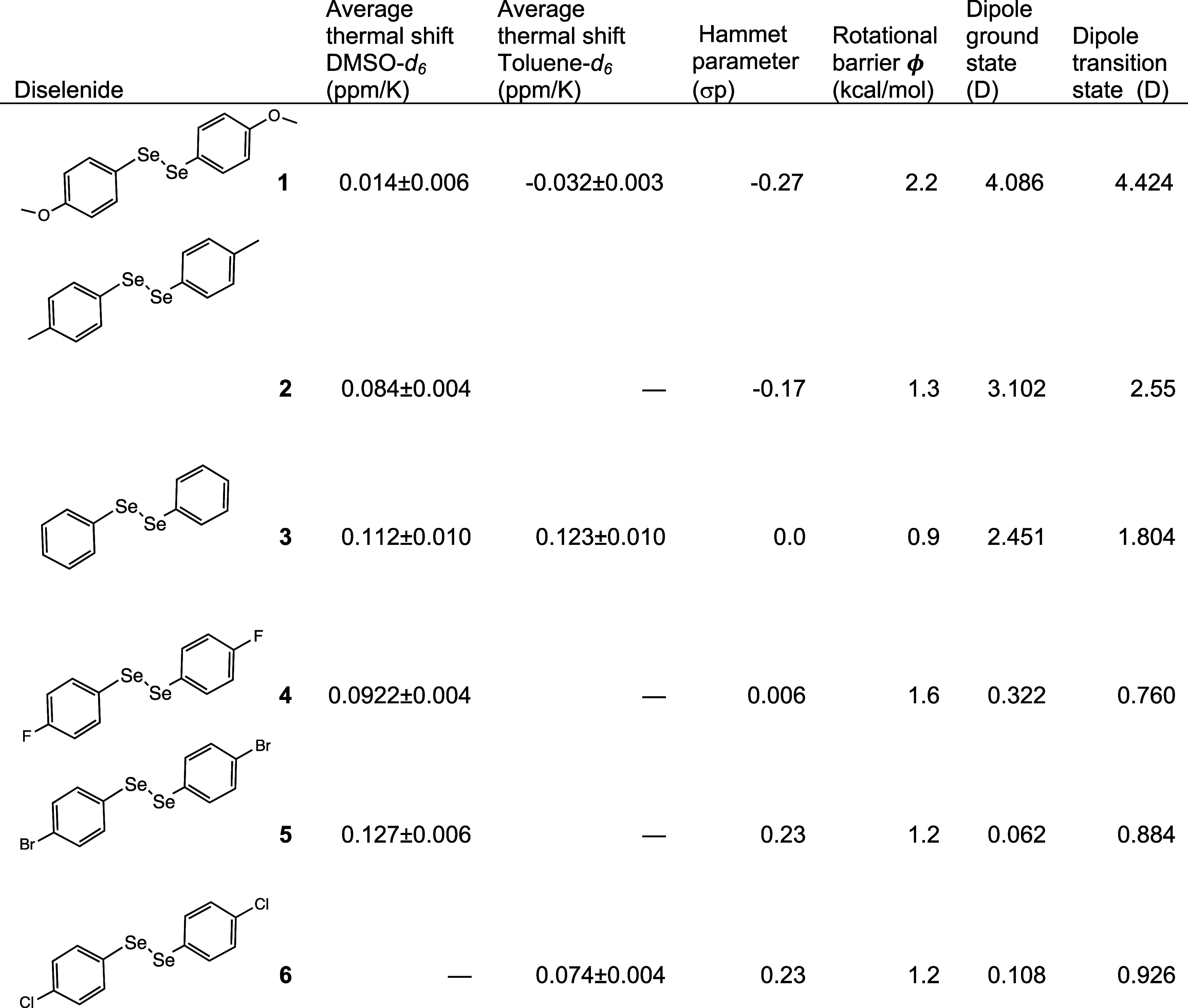
Thermal ^77^Se NMR Shifts of Selected Diaryl Diselenides

Previously, chemical shifts of several ppm over a temperature range of up to 100 °C have been reported in diaryl diselenides (0.1–0.4 ppm/K)^27^ and dialkyl diselenides (0.1–0.3 ppm/°C).^28^ Shape distortions and line broadening have been noted as heat is applied in variable temperature studies.^27^ Our work on both diaryl diselenides and dialkyl diselenides showed chemical shifts of 0.066–0.1286 ppm/K and fit within the reported ranges. In addition, peak broadening was noted at elevated temperatures.

What initially brought our attention to this question were the massive shifts in ^77^Se NMR peaks that occur as diaryl and dialkyl diselenides are heated *in situ*, compared to that of monoselenides (0.025 ppm/K for CH_2_Se).^27^ Furthermore, the magnitude of the shift is highly dependent on the diselenide substituents.^28^ We developed several hypotheses to explain these large chemical shifts and sought further evidence to confirm or refute the ideas.

### Hypothesis 1: Rapid Equilibrium and Solute–Solute Interactions

**Scheme 1:**
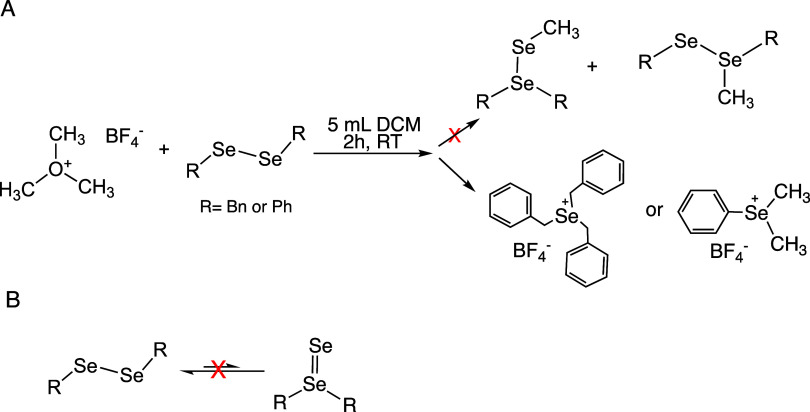
(A) Reaction between Meerwein’s Salt and Diaryl Diselenide as an Attempt to Chemically Prove the Fast Equilibrium Hypothesis; Crystal Structures of Products Achieved Shown in Figure S1; (B) Proposed Mechanism Explored in Hypothesis 1, Which Was Disproved

One hypothesis postulated that diselenides may be in a temperature-dependent equilibrium with an R_2_Se=Se species (Scheme 1). The relative equilibrium position of RSe-SeR to R_2_Se=Se would cause the temperature-dependent shift, and a rapid equilibrium would cause only one peak seen in the NMR given the slow ∼30s relaxation times of ^77^Se nuclei.^29^ This may even be a reactive intermediate in the decomposition of diselenides to mono- and triselenides.^30^ These mechanisms have been proposed in disulfides in the past.^31−33^ As shown in Scheme 1, this double-bonded intermediate was not isolated by using *in situ* heated NMR.

Ph_2_Se_2_ and Bn_2_Se_2_ were also examined by variable-temperature solution IR in dioctyl ether to study this possible transformation on a faster time scale. In both cases, a Se–Se stretch was identified at 850 cm^–1^(Figure S3). No significant change in the IR was observed on heating to 160 °C. Also, between −20 and 50 °C, the UV–vis did not change (between 200 and 500 nm, Figure S3).

One more study was performed to provide evidence of a double-bonded structure. In reactions of Meerwein’s salt (1) with Me_2_Se_2_, methylation of one of the Se is seen to give MeSeSeMe_2_.^34^ If a rapid equilibrium was occurring between RSeSeR and R_2_Se=Se, then a reaction of dibenzyl or diphenyl diselenide with Meerwein’s salt would result in a combination of (2) and (3) (Scheme 1). We attempted this reaction several times with both Ph_2_Se_2_ and Bn_2_Se_2_. Instead of methylated diselenides, [Bn_3_Se^+^][BF_4_^–^] and [PhMe_2_Se^+^][BF_4_^–^] were isolated (crystal structures in Figure S1).

Finally, DFT was used to calculate the ground state energy of each structure (Table S1). The free energy was 16.5 and 15.2 kcal/mol greater for the double-bonded isomers of Ph_2_Se_2_ and Bn_2_Se_2_ respectively over their standard conformations. These energy differences are quite large, resulting in a population ratio of 10^–12^:1 in favor of the single-bonded form for both species at room temperature. This massive difference in energy makes it unlikely that a double-bonded species contributes to the large thermal NMR shift.

Solute–solute interactions were considered as previous work using ^125^Te NMR to evaluate concentration effects showed that ditellurides have small shifts downfield with increases in concentration.^35^ A concentration series was studied in both (*p*-CH_3_PhSe)_2_ and Ph_2_Se_2_ in DMSO-*d*_6_ (0.001 M–0.1 M) (Figure S4), and no substantial ^77^Se NMR shifts were observed (solvent DMSO-*d*_6_). Therefore, it was concluded that **2** and **3** do not suffer from solute–solute interactions that alter the ^77^Se NMR shifts.

### Hypothesis 2: Shielding Effects

Perhaps the electron environment around diselenides makes them particularly prone to deshielding with increasing temperature. If this were the case, then the thermal shift should be exquisitely sensitive to the chemical environment and the presence of electron-withdrawing and electron-donating substituents. Therefore, the magnitude of the thermal shift should correlate with the Hammett parameter (*para-*substituents) and electron donating or withdrawing properties of these substituents. Deshielding may make the electron density of the Se more prone to thermal changes, while shielding would have the opposite effect. Ultimately, it was hypothesized that the electron-withdrawing abilities of the Hammett parameter substituents dictate the electron density around the selenium and thus control the thermal shift of the molecules.

A procedure adapted from Braga et al. was used to synthesize five substituted diselenides.^16^ The identity and purity of the diselenides were confirmed using ^1^H and ^77^Se NMR as well as thin layer chromatography. Silica gel chromatography was performed for purification, when necessary. All samples were heated as 0.017 M solutions in DMSO-*d*_6_ or toluene-*d*_8_, and ^77^Se NMR was taken at several temperature points. Each substituted diselenide had a downfield shift (ppm/K) and they are ordered by shift magnitude accordingly: Br > H > CH_3_ > Cl > *F* > OCH_3_. **6** was unstable in the DMSO-*d*_6_, so instead toluene-*d*_8_ was used as the NMR solvent.

When evaluating these molecules at room temperature there is a linear trend between chemical shift and Hammett parameter,^8^ so it was hypothesized that the thermal shift should also have a linear trend if this was the appropriate explanation. Comparing the Hammett parameter to the thermal shift shows only a mild correlation (Table 1). There was also little correlation between the magnitude of the temperature-dependent shift and the initial chemical shift, which is a much more direct measure of the electron density (Figure 1). An alternative, or at least more nuanced, explanation for the large thermal shifts of the diselenides was needed.

### Hypothesis 3: Solvent Effects

The magnitude of the chemical shift change is dependent on the identity of the NMR solvent, and the effect was not consistent across all diselenides studied. In the previous work by Lardon et al., this effect was not observed, since neat melts were employed.^8^ For example, Ph_2_Se_2_ in DMSO-*d*_6_ undergoes a temperature-dependent chemical shift change of 0.112 ppm/K, while in toluene-*d*_8_ the value is slightly larger at 0.123 ppm/K. In contrast, the *p*-methoxyphenyl substituted diselenide shifted 0.0142 ppm/K in DMSO-*d*_6_, but in toluene-*d*_8_ the shift had the opposite sign at −0.0323 ppm/K. This difference, especially in **1**, led to the hypothesis that the shifts we see in ^77^Se NMR may be related to molecular polarity and solvation effects. Thus, solvent effects were deemed to be an important hypothesis to explore.

Solvent effects are often discussed in NMR, however, and the reasoning behind them is often opaque. Odom et al. proposed that the solvent polarizability plays a role in the resulting ^77^Se NMR chemical shift.^5^ They based this hypothesis on ^77^Se NMR studies of (CH_3_)_2_Se in a series of solvents including DMSO-*d*_6_ and CDCl_3_ to compare the resulting chemical shift values.^5^ In their research characterizing (CH_3_)_2_Se as an internal standard, they found that each solvent gives a distinct chemical shift (no standard is present). Further, there was a 0.025 ppm/K shift downfield when the sample was heated from 223 to 323 K in CDCl_3_.^5^ Our study instead uses DMSO-*d*_6_ which allows for higher temperatures to be achieved compared to CDCl_3_ but is also more polar than other NMR solvents and can act as a Lewis base.

### Hypothesis 4: Barriers to Rotational Movement

Rotational motion through different conformers may be the source of the thermal shifts; elevated temperatures cause the molecules to spend more time in twisted conformations that have NMR shifts differing from those of the ground state position. Such a hypothesis may also explain the solvent dependence of the thermal shifts, as some conformers are more polar than others.

**Figure 3:**
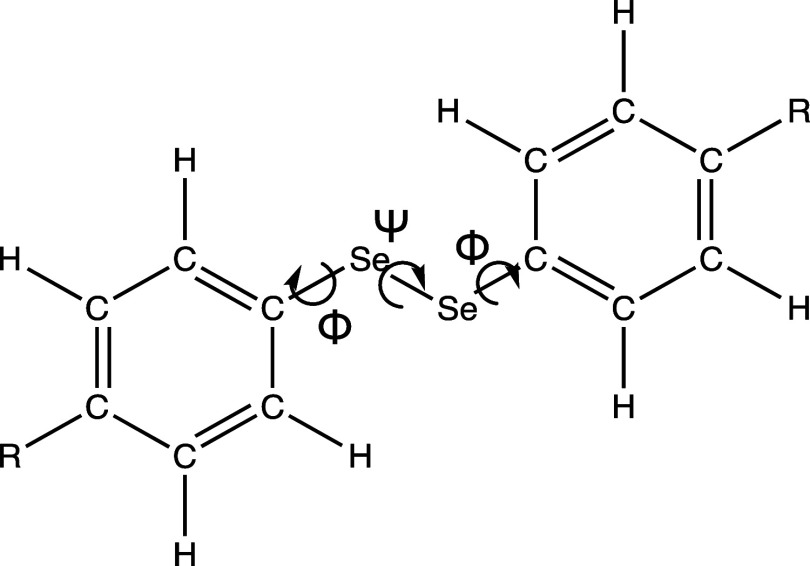
Adapted from the work of Orian et al. Bond rotations in the diaryl diselenide.

**Figure 4:**
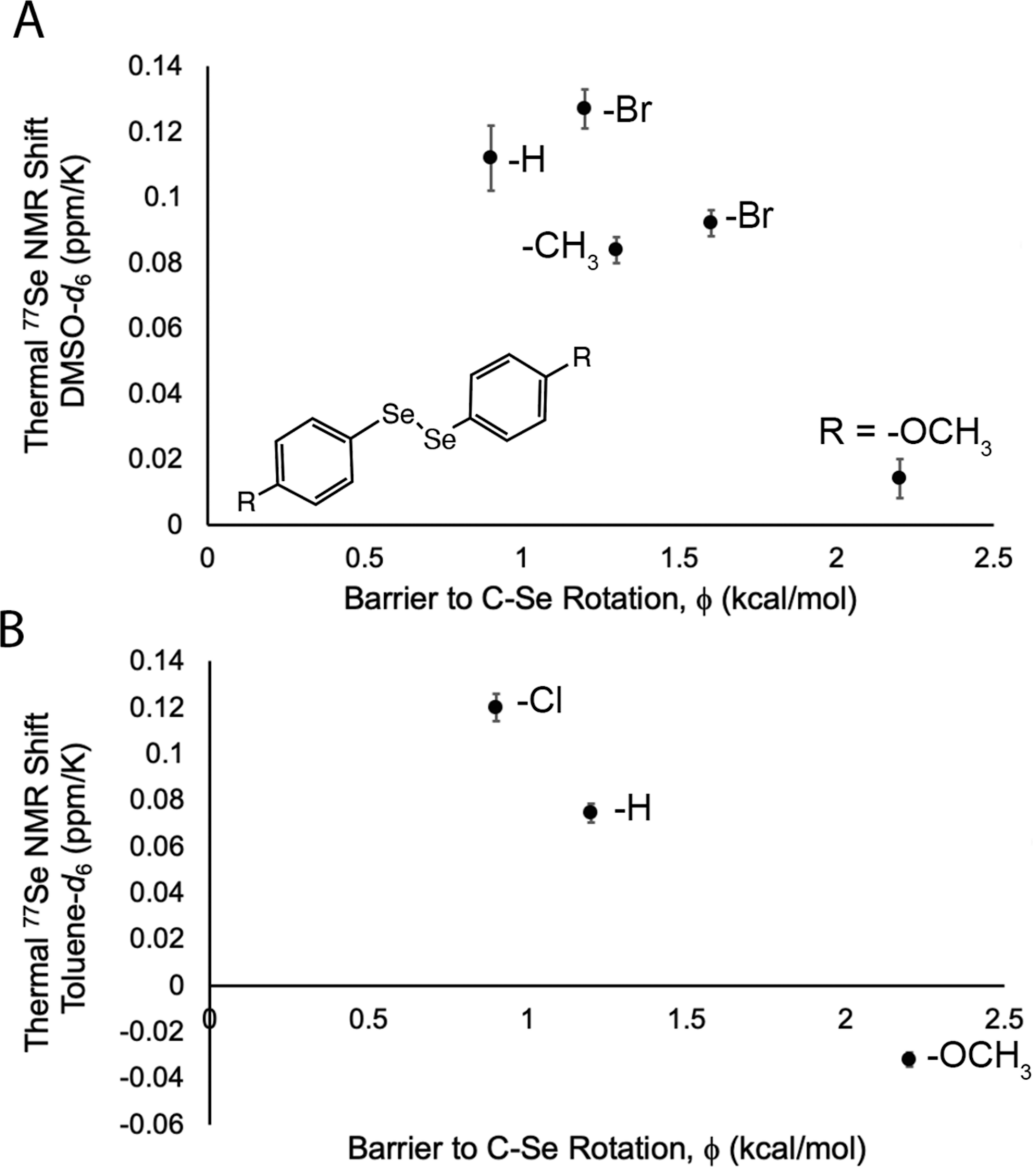
Scatter plots showing (A) ppm/K versus barrier height energy in five diselenides heated in DMSO-*d*_6_. (B) ppm/K vs barrier height energy in three diselenides heated in deuterated toluene. There is no strong relationship between the parts per million per kilogram shift and the barrier height energy or dipole energy.

The dihedral rotation, Φ (Figure 3), about the C—Se bond can be a possible explanation for the thermal change in ^77^Se NMR shifts. In a previous publication, Orian et al. calculated the gas-phase rotational barriers of the C–Se dihedral (Φ) of several diaryl diselenides and found the open conformer (where both the rings are facing one another) to be the minimum and the closed conformer to be the transition state (Figure 3). The barrier to this rotation had a relationship with the functional group in the *para* position following the Hammett series and ranging in energy from 0.8 to 2.6 kcal/mol.^36^ These energies are not much larger than room temperature thermal energy of 0.58 kcal/mol, and are consistent with the observation that that the phenyl rings of diselenides rotate freely in solution.^37^ Plotting the calculated gas-phase Φ barriers against the observed thermal shifts in DMSO and toluene does not yield a good correlation (Figure 4). Unfortunately, a poor correlation between the Φ and thermal ^77^Se NMR shift is not a satisfactory reason to rule this hypothesis out.

If Φ rotation of the phenyl rings is important, then dialkyl diselenides should not have large thermal shifts since the dialkyl diselenides do not have this rotation. However, the thermal shift for didodecyl diselenide was measured to be 0.192 ± 0.015 ppm/K, in 1,2-dichlorobenzene-*d*_4_, which is similar in magnitude to that of the diaryl diselenides (Figure S12 and S13). The thermal shift for didodecyl selenol was much smaller at 0.022 ppm/K in 1,2-dichlorobenzene-*d*_4_ (Figure S13). The large thermal shift is not unique to diaryl diselenides, and therefore, Φ rotation can be ruled out as a major contributor.

Rotation about the Se–Se bond, Ψ, is more hindered than Φ rotation. The energy difference between different conformations was calculated for **1 and3**. Based on previous work, three points were considered at approximately Ψ = 90° (skewed conformation) and Ψ = 0°, 180° (*syn* and *anti*-coplanar conformations) which correspond to a local minimum and two maxima in the rotation of the Ψ dihedral.^36^ Both molecules showed a gas phase energy difference of ∼7 kcal/mol (Ψ = 0°) and a lower energy difference of ∼4 kcal/mol for Ψ = 180°. Both energies are low enough that the molecules can freely rotate in solution at room temperature within the NMR time scale, *albeit* at a lower frequency than the Φ rotation.^38^

**Figure 5:**
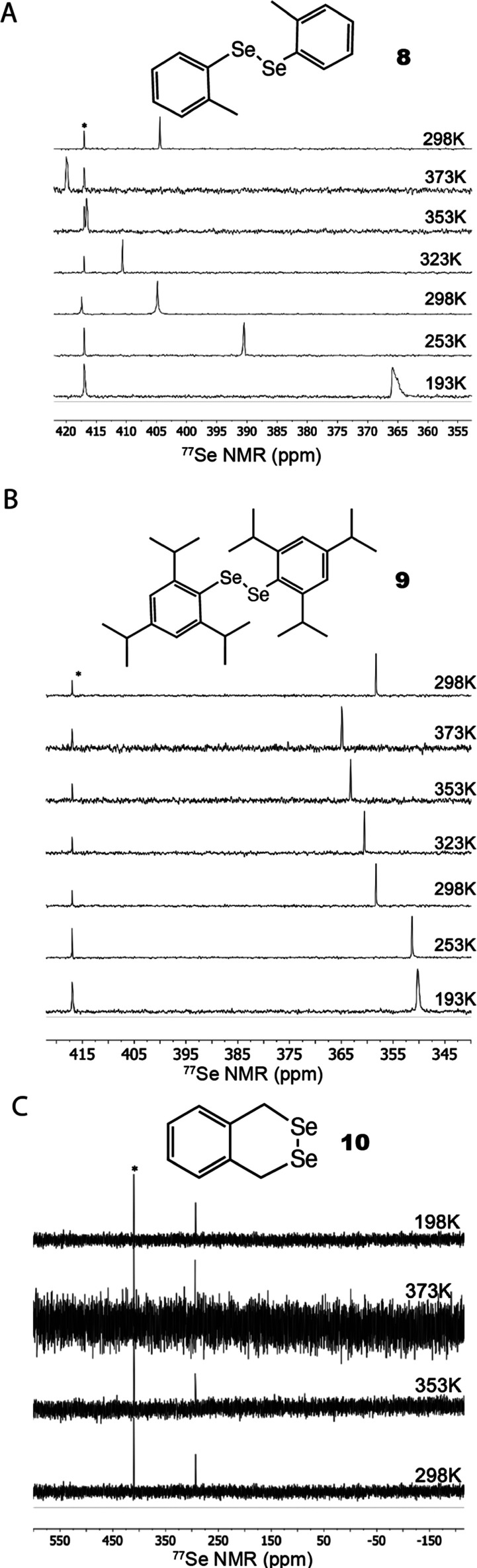
^77^Se NMR of sterically hindered diselenides cooled and heated to temperatures between 193 and 373 K. No splitting of peaks was observed, and chemical shifts always returned to their original positions upon cooling to 298 K. (A) 1,2-di-o-tolyldiselenide (toluene-*d*_8_) (8), (B) 1,2-bis(2,4,6-triisopropylphenyl)diselenide (toluene-*d*_8_*)* (9), and (C) 1,4-dihydrobenzo[*d*][1,2]diselenine (DMSO-*d*-_6_) (10). * marks the internal standard Ph_2_Se. Full spectra are available in Figure S7 and ^1^H of bis(2,4,6-triisopropylphenyl)diselenide is available in Figure S8. Figure S11 plots temperature-dependent chemical shifts versus temperature.

To provide more evidence that rotation around Ψ is the source of the large thermal shifts of diaryl and dialkyl diselenides, we synthesized and characterized a small series of diselenides that experience increasing hindrance to rotation (Figure 5, Table 2). These included 1,2-di-*o*-tolyldiselenide (**8**), 1,2-bis(2,4,6-triisopropylphenyl)diselenide (**9**), and 1,4-dihydrobenzo[*d*][1,2]diselenine. In toluene, 1,2-di-*o*-tolyldiselenide experienced a thermal shift of 0.219 ± 0.011 ppm/K indicating that the *o-*methyl groups do not provide steric hindrance for rotation around the Se–Se position. This is likely because the barrier for Φ rotation around the Se–C bond, giving a “barn door” open vs closed configuration of the phenyl groups, is very low energy. Therefore, the protruding methyl groups can easily adopt a parallel configuration and do not sterically interfere with one another. In contrast, 1,2-bis(2,4,6-triisopropylphenyl)diselenide experienced only a 0.086 ± 0.004 ppm/K thermal shift. The isopropyl groups protrude out of plane with the phenyl groups and can sterically interfere with each other even if the phenyl groups are in “barn door open” configuration. Lastly, 1,4-dihydrobenzo[*d*][1,2]diselenine (**10**) is locked out of Ψ rotation around the Se–Se bond and consequently the thermal shift was nearly undetectable and only 0.009 ± 0.001 ppm/K. Molecules that have hindered rotation around the Se–Se bond show little or no thermal shift indication that rotation around the Se–Se bond is the source of the large thermal shift often seen in diselenides.

**Table 2:**
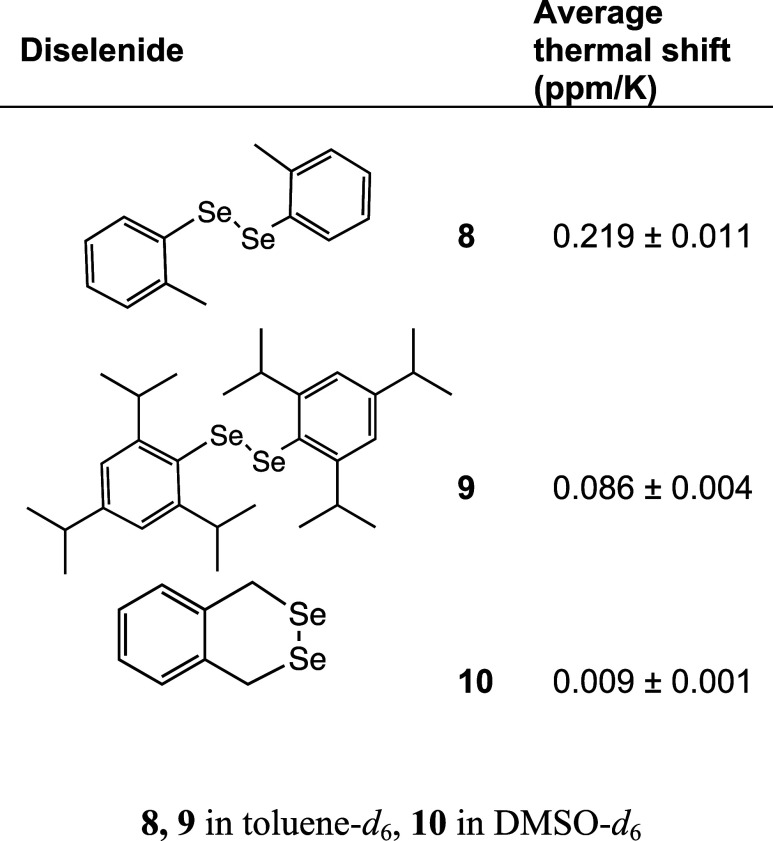
Thermal ^77^Se NMR Shifts of Rotationally Hindered Diselenides

### Hypothesis 5: Solvent Effects on Se–Se Rotations

Based on the above experiments, we are quite confident that the source of the large thermal ^77^Se NMR shifts of many diselenides comes from rotation about the Se–Se bond. What we cannot yet describe is why some shift more than others. There are two convoluted factors: (1) in some molecules the twisted conformer may have a larger difference in NMR shift than the minimum energy conformer and (2) transition state theory suggests processes with a larger barrier are more sensitive to population differences upon heating.

For example, the energy difference between the skewed and coplanar structures was calculated for **1** (*anti*) and **3** because they represent molecules with a small thermal shift and a large thermal shift in the experiments. **3** has a larger energy difference (4.76 kcal/mol in the gas phase) than **1** (4.45 kcal/mol in the gas phase), supporting the idea that perhaps larger barriers are correlated with larger thermal shifts.

Sampling twisted conformations may also explain some of the solvent dependence. Calculations indicate the energy difference between skewed and coplanar geometries for **3** (4.51 kcal/mol in toluene, 4.15 kcal/mol in DMSO) compared to **1** (4.33 kcal/mol in toluene and 3.86 kcal/mol in DMSO is magnified in more polar solvents. The calculations are consistent with the experiment: using DMSO results in a larger thermal shift difference between **1** and **3** than in toluene (Table 3 and Table 4). Bartz et al. reported that there is a small solvent-dependent shift in disubstituted diselenides through a study of eight different deuterated solvents including DMSO-*d*_6_.^39^ Through computational and synthetic methods they reported that DMSO-*d*_6_ and other polar solvents caused shielding of ^77^Se chemical shifts at room temperature.^39^ Other reports show that both DMSO and toluene have small magnetic susceptibilities with heating —with an average chemical shift of 0.004 ppm seen when heating from 0 to 70 °C.^40^

**Table 3:** Dipoles, μ, (Debye), and Electronic Energies (kcal/mol) for the Investigated Structures of Ph_2_Se_2_^a^

	Ψ = 0°		Ψ = 90°		Ψ = 180°	
	dipole (D)	Δ*E* (kcal/mol)	dipole (D)	Δ*E* (kcal/mol)	dipole (D)	Δ*E* (kcal/mol)
gas	3.6795	7.20	2.451	0.00	0.005	4.76
toluene			2.805	–8.22	0.005	–3.71
DMSO			3.211	–5.60	0.006	–1.45

a Calculation on the Ph_2_Se_2_ structures with Y = 0° resulted in a structure at higher energy; therefore, the investigation was not pushed further. Level of theory (SMD)-B3LYP/cc-pVTZ.

**Table 4:** Dipoles, μ, (Debye), and Electronic Energies (kcal/mol) for the Investigated Structures of Ph_2_Se_2_-(*p*OCH_3_)_2_^a^

	Ψ = 90°, R = syn		Ψ = 90°, R = anti		Ψ = 180°, R = syn		Ψ = 180°, R = anti	
	dipole (D)	Δ*E* (kcal/mol)	dipole (D)	Δ*E* (kcal/mol)	dipole (D)	Δ*E* (kcal/mol)	dipole (D)	Δ*E* (kcal/mol)
gas	5.156	0.02	4.086	0.00	2.330	4.45	0.032	4.44
toluene	5.094	–8.96	4.800	–8.99	2.774	–4.66	0.024	–4.67
DMSO	7.306	–8.09	5.636	–7.85	3.407	–3.98	0.026	–3.99

a Level of Theory (SMD)-B3LYP/cc-pVTZ.

However, these energy differences to barrier height for the two species (∼0.3 kcal/mol) are very similar to that of the solvent changes (0.3–0.4 kcal/mol), suggesting these effects should be of similar magnitude, which they are not. The limitations of the calculations may not accurately reflect the physical reality, and these numbers should not be overanalyzed.

The calculations for barrier height also fail to explain why **1** has a very slight positive thermal shift in toluene, given that the calculations suggest that there is still a significant 3.86 kcal/mol barrier. Therefore, it is likely that the temperature dependence of the ^77^Se NMR shifts is influenced by the chemical shift dependence of the C–Se–Se-C dihedral sampling and, thus, conformational sampling.

While there have been recent advances in the calculations of ^77^Se NMR chemical shifts, the calculations are not ready to reliably calculate differences of just a few ppm.^41^ Correctly predicting the thermal shifts of diselenides would be an excellent target.

## Conclusions

Thermal changes in the ^77^Se NMR chemical shifts were measured for several substituted aryl disulfides and an alkyl disulfide, and were found to range from −0.032 to +0.219 ppm/K depending on the species and solvent. Five hypotheses were presented and examined to explain the large thermal shifts seen.

The most likely explanation is that the temperature changes the sampling of rotational conformations around the Se–Se bond (Ψ). The barrier to rotation from α Ψ ∼ 90° through 180° was calculated to be ∼4 kcal/mol in simple unencumbered diselenides and so is an appropriate scale to see such an effect at moderate temperatures. Locking out the rotation around the C–Se–Se-C bond using bulky and tethered diselenides reduced and even eliminated the thermal shift, further corroborating the hypothesis.

Calculations of the rotation of the phenyl ring around the C–Se bond (Φ) show a small barrier to rotation (0.8–2.6 kcal/mol), on par with the thermal energy at room temperature. Plotting the calculated barriers did not show a good correlation with the experimental data, and so the Φ rotation was deemed less important to the observed thermal shifts.

Other hypotheses were examined and excluded. No evidence for a fast equilibrium with a R_2_Se=Se species was found; the IR and UV spectra did not shift with temperature, nor could such a species be captured through reaction with Meerwein’s salt. Unlike ditellurides,^42^ the NMR shifts were not concentration dependent, suggesting there are no significant solute–solute integrations that might explain the large thermal ^77^Se NMR shifts.

A hypothesis of simple electronic electron-withdrawing effects altering the susceptibility of the shielding environment to temperature was also excluded. There was no easy correlation of the magnitude of the thermal shifts with the Hammett parameter of *para*-substituted diaryl diselenides, likely because the magnitude of the thermal shift involves both the size of the energetic barrier to rotation and the chemical shift difference between the twisted and ground state conformers.

Changing the solvent between DMSO and toluene changed the magnitude of the thermal ^77^Se NMR shifts of several aryl diselenides and, in one case, reversed its direction. While this likely has to do with the differing polarity of the low and high-energy conformers, DFT calculations that included solvent dielectric did not correlate well with the experimental results. The calculations likely need the inclusion of explicit solvent molecules to explain the experimental results, and this is an area for further research. Moving forward, the phenomenon should also be studied again in water, which is more biologically relevant, but we should also consider the localized polarity environments when considering the rotational flexibility of diselenides on and in protein structures. As well, the predictive power of calculations of ^77^Se NMR shifts still remain

Since there is not yet a predictive way to include the role of solvent polarity, the interpretation of rotational flexibility is qualitative. A substantial thermal ^77^Se NMR shift should be considered a sign of rotational flexibility, but a very small one does not necessarily mean there is no rotational flexibility.

The study demonstrates that the ^77^Se NMR thermal shift is a way to qualitatively characterize the rotational flexibility about the Se–Se bond in diselenide-containing species. This is another characterization handle to study these diselenide functionalities in proteins and stimuli-responsive polymers.

## Supporting Information Available

Crystal structures, DFT calculations, UV–vis spectra, IR spectra, ^77^Se NMR spectra of concentration studies and cooled NMR studies, ^1^H NMR spectra of precursors, and ^77^Se NMR spectra of heated dialkyl diselenides (PDF)

## Abbreviations

Ph_2_Se_2_: diphenyl diselenide
Bn_2_Se_2_: dibenzyl diselenide
DDSeH: didodecyl diselenol
DD_2_Se_2_: didodecyl diselenide
RSeH: selenol
SeO_4_^2–^: selenate
NMR: nuclear magnetic resonance
PID: proportional integrative derivative controller
IR: infrared spectroscopy
UV–vis: ultraviolet visible spectroscopy
DFT: density functional theory
μ: dipole
Φ: C–Se bond rotation
Ψ: Se–Se bond rotation
