# Supplementary material for: Five Hypotheses on the Origins of Temperature Dependence of 77Se NMR Shifts in Diselenides
Source: Inorg Chem. 2024 Jun 14;63(26):12063–72. doi: 10.1021/acs.inorgchem.4c01025 (PMC11220761; doi:10.1021/acs.inorgchem.4c01025)
Supplement: Supplementary file 1 — ic4c01025_si_001.pdf [file ic4c01025_si_001.pdf]

Supplemental information: Five hypotheses on the origins of temperature dependence of ^{77}Se NMR shifts in diselenides

*Alexandra C. Koziel[§], Marco Bortoli[†], Matthew Tremblay[§ †], Yilun Zhao[§], Laura Orian[†], Zhongyue J. Yang[§], Nathan D. Schley[§], Janet E. Macdonald**

[§]Department of Chemistry, Vanderbilt University, 1234 Stevenson Center Lane, Nashville, TN, 37240, United States

[†] Dipartimento di Scienze Chimiche, Università degli Studi di Padova, Via Marzolo 1, 35131 Padova, Italy

*janet.macdonald@vanderbilt.edu

Table of Contents:

Crystal structures **S2**

DFT calculations **S2**

In situ IR **S3**

UV-Vis Spectroscopy **S3**

Concentration Studies **S4**

Full thermal ^{77}Se NMR **S5**

^{1}H NMR for *para* substituted diselenides **S6**

^{1}H and ^{77}Se NMR of sterically bulky molecules **S7**

Temperature dependent chemical shifts **S8**

Dialkyl diselenide ^{77}Se thermal shift **S11**

Coordinates of DFT structures **S12**

Polarity analysis **S17**

References **S17**

Crystal Structures and DFT Calculations

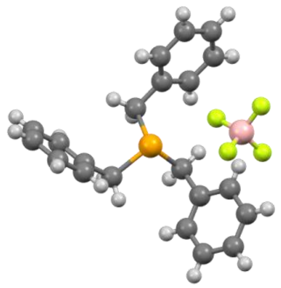

$[Bn_3Se][BF_4]$

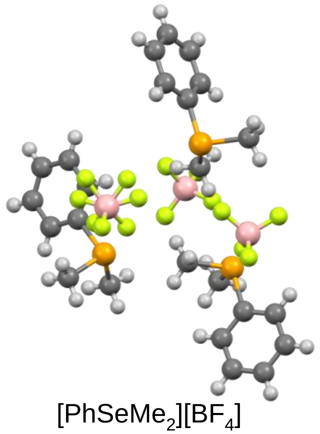

$[PhSeMe_2][BF_4]$

Figure S1. Proposed structures and formula from single crystal X-ray crystallography of the products of the reactions with Meerwein's salt. A) Proposed structure of the reaction product of Bn_2Se_2 with Meerwein's salt. B) Proposed structure of the reaction product of Ph_2Se_2 with Meerwein's salt.

Table S1. DFT calculations showing the energy differences and population ratio between the diphenyl diselenide and the proposed double bonded species.

Compound	ΔG (kcal/mol)	Ratio at 25˚C
Diphenyl diselenide	0.0	
Double bonded diphenyl diselenide	16.5	8.4×10^{-13}

Spectroscopy for Hypothesis 1

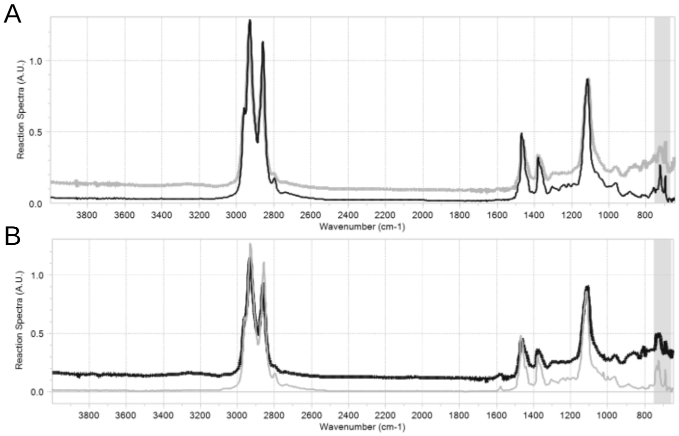

Figure S2. *In situ* heated IR of A) Ph_2Se_2 and B) Bn_2Se_2 in dioctyl ether at room temperature (grey) and 160ºC (black).

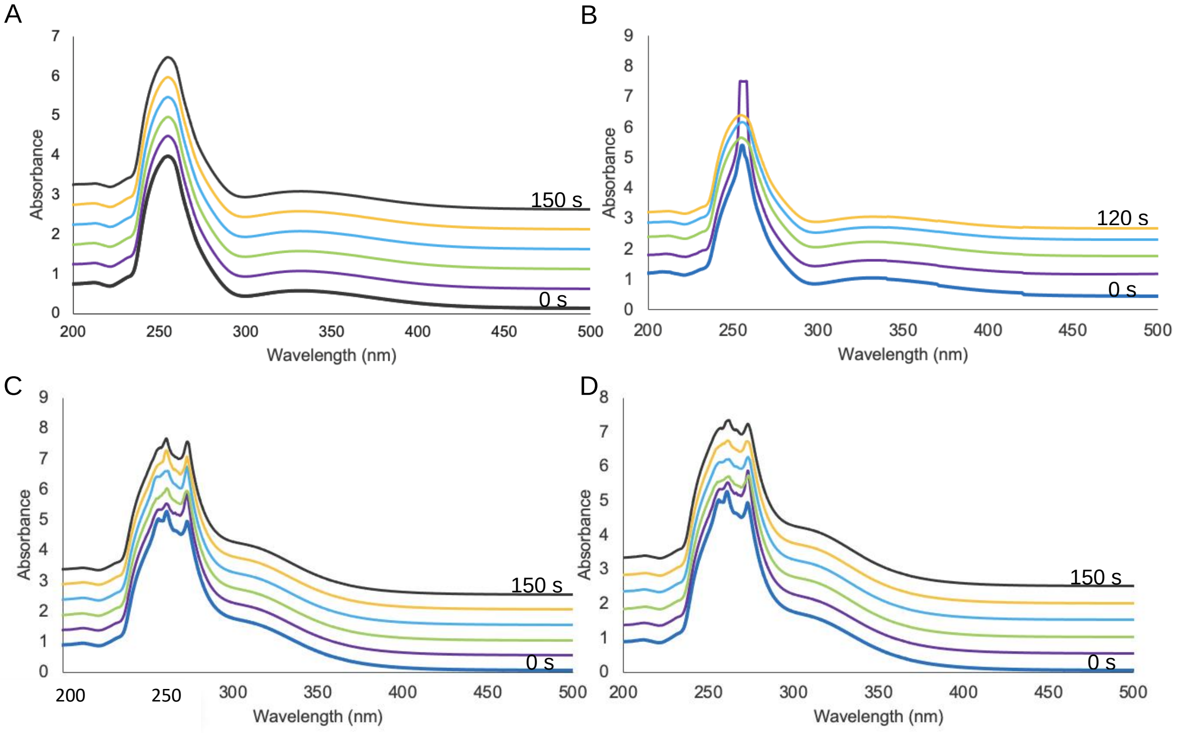

Figure S3. UV-Vis of A) Ph_2Se_2 heated in chloroform to 50ºC B) Ph_2Se_2 cooled in chloroform to -20ºC C) Bn_2Se_2 heated in chloroform to 50ºC D) Bn_2Se_2 cooled in chloroform to -20ºC. All spectra were run in 30 second increments starting at 0 seconds.

Concentration Studies

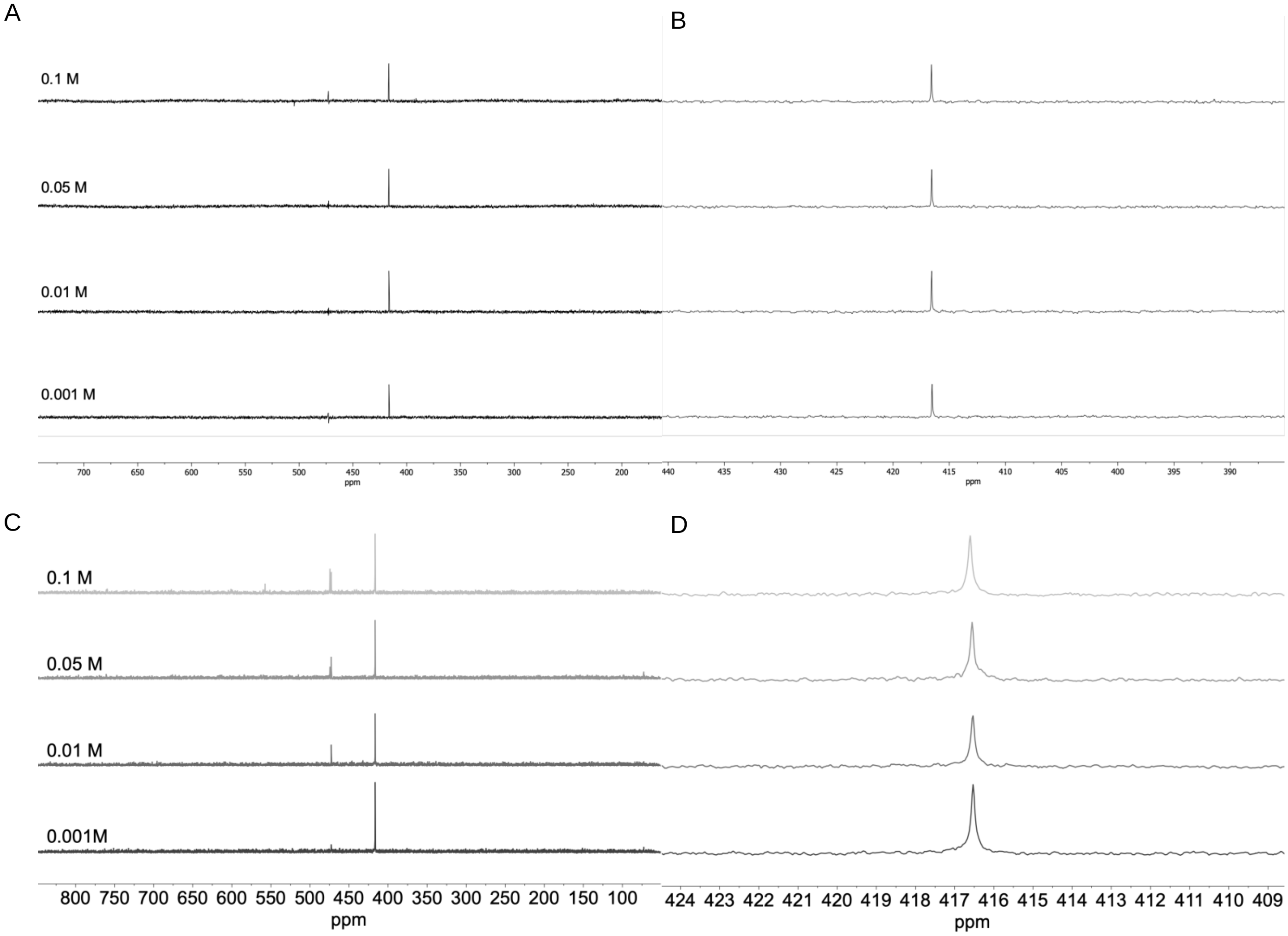

Figure S4. Concentration studies run at molarities between 0.001 and 0.1 M in A and B) anisole substituted diselenide and C and D) toluene substituted diselenide. No movement in the chemical shift was observed. A diphenyl selenide is present at 473ppm as an internal standard and these spectra were performed in DMSO-d_6.

Full Heated ^{77}Se NMR Spectra

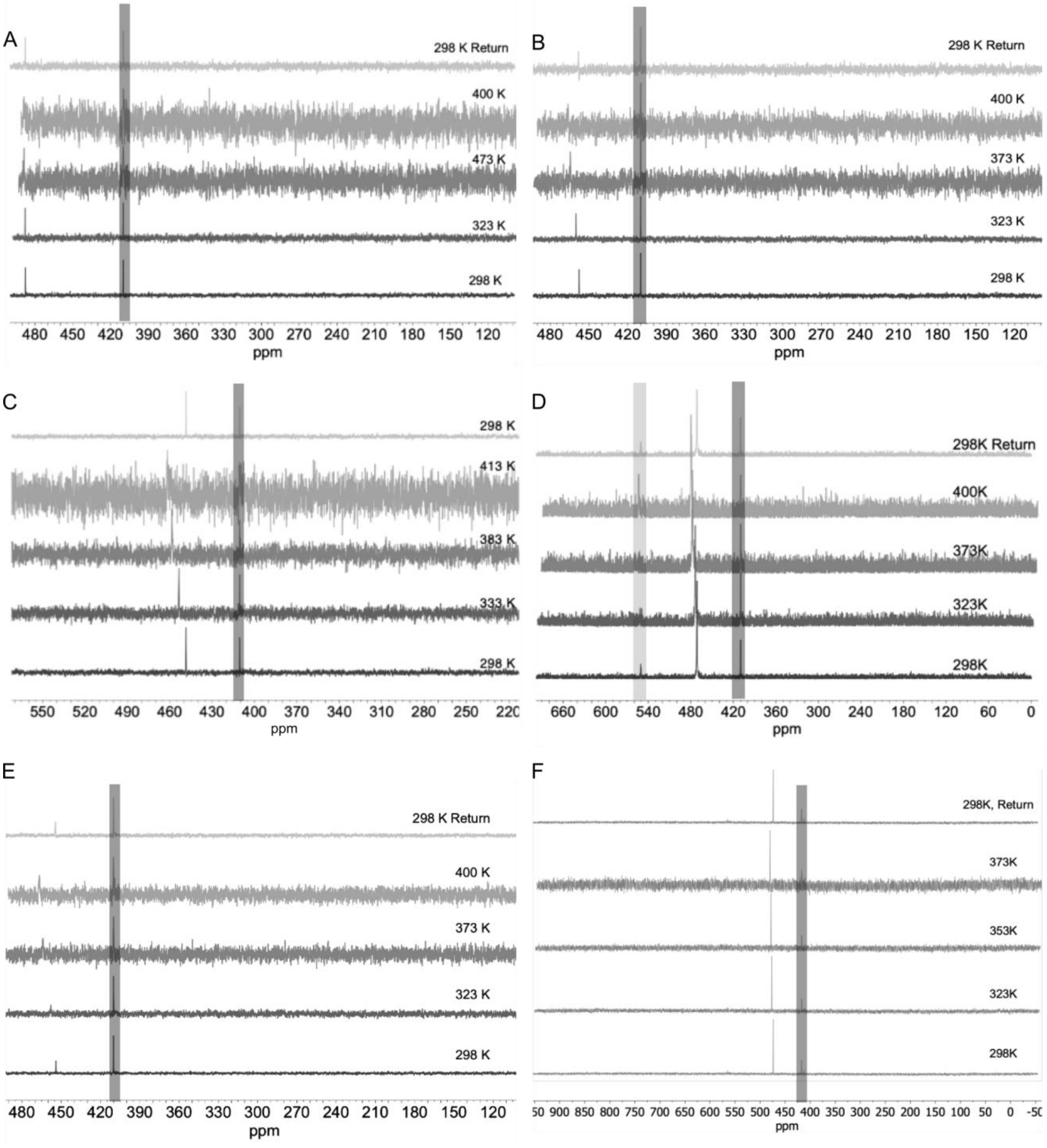

Figure S5. Heated ^{77}Se NMR with Ph_2Se internal standard of A)1,2-bis(4-methoxyphenyl)diselane B) 1,2-di-*p*-tolyldiselane C) diphenyl diselenide D) 1,2-bis(4-fluorophenyl)diselane E) 1,2-bis(4-bromophenyl)diselane F) 1,2-bis(4-chlorophenyl)diselane (in toluene-d_8). All NMR preparations were run in DMSO-d_6 unless otherwise noted.

^{1}H NMR of *para* Substituted Diselenides

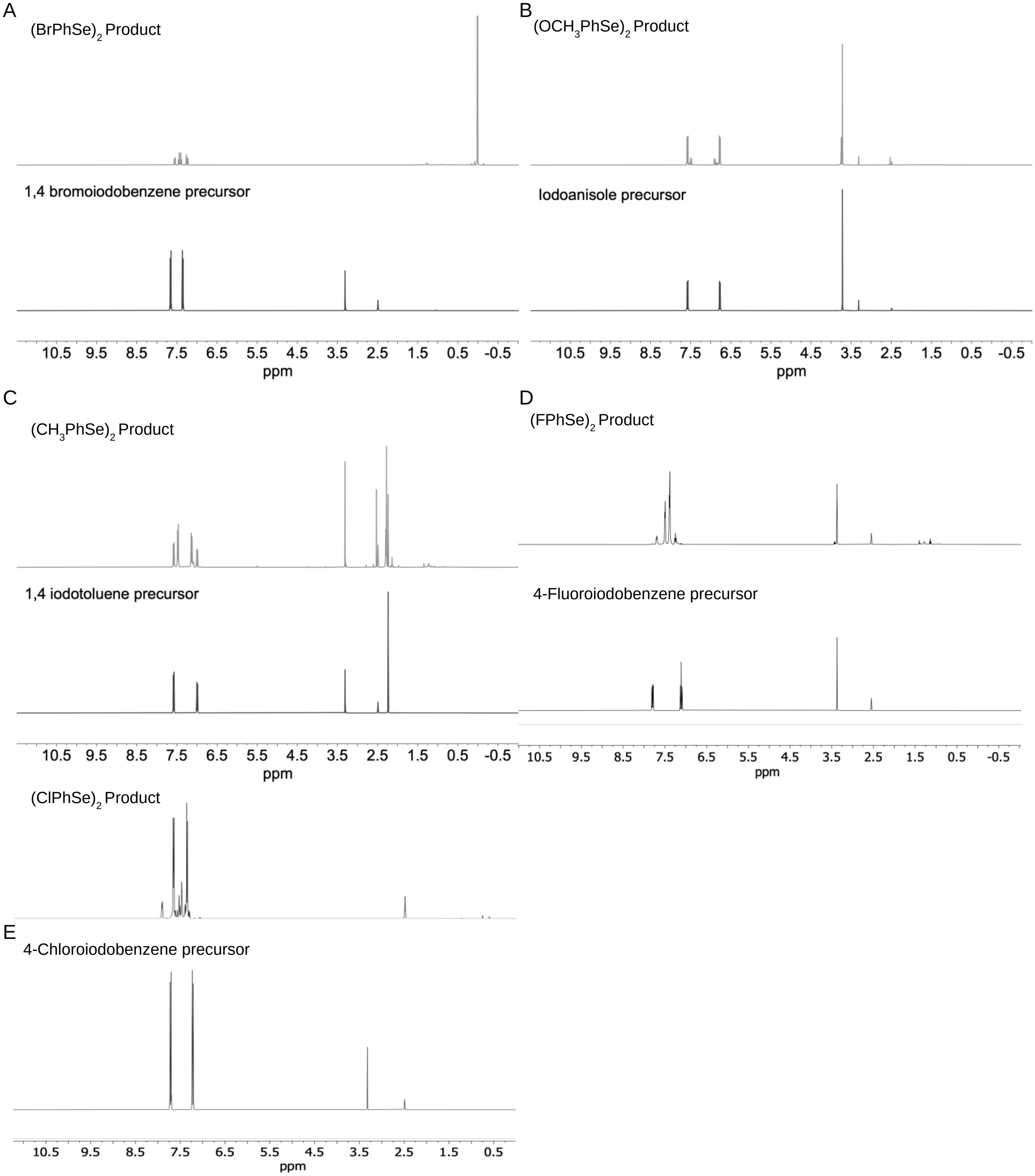

Figure S6. ^{1}H NMR comparing precursor to product of five synthesize *para* substituted diselenides. A) $(BrPhSe)_2$ B) $(OCH_3PhSe)_2$ C) $(CH_3PhSe)_2$ D) $(FPhSe)_2$ E) $(ClPhSe)_2$. All spectra performed in $CDCl_3$.

^{1}H and ^{77}Se NMR of sterically bulky molecules

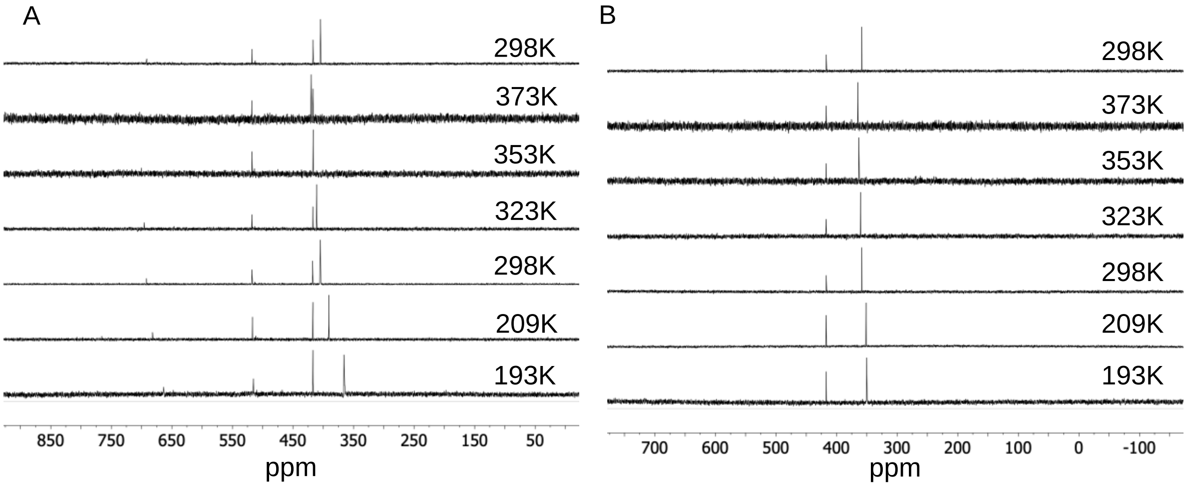

Figure S7. (toluene-d_8) Full ^{77}Se spectra of cooled and heated experiments evaluating thermal movement of chemical shifts in A)1 ,2-di-*o*-tolyldiselane and B) 1,2-bis(2,4,6-triisopropylphenyl)diselane.

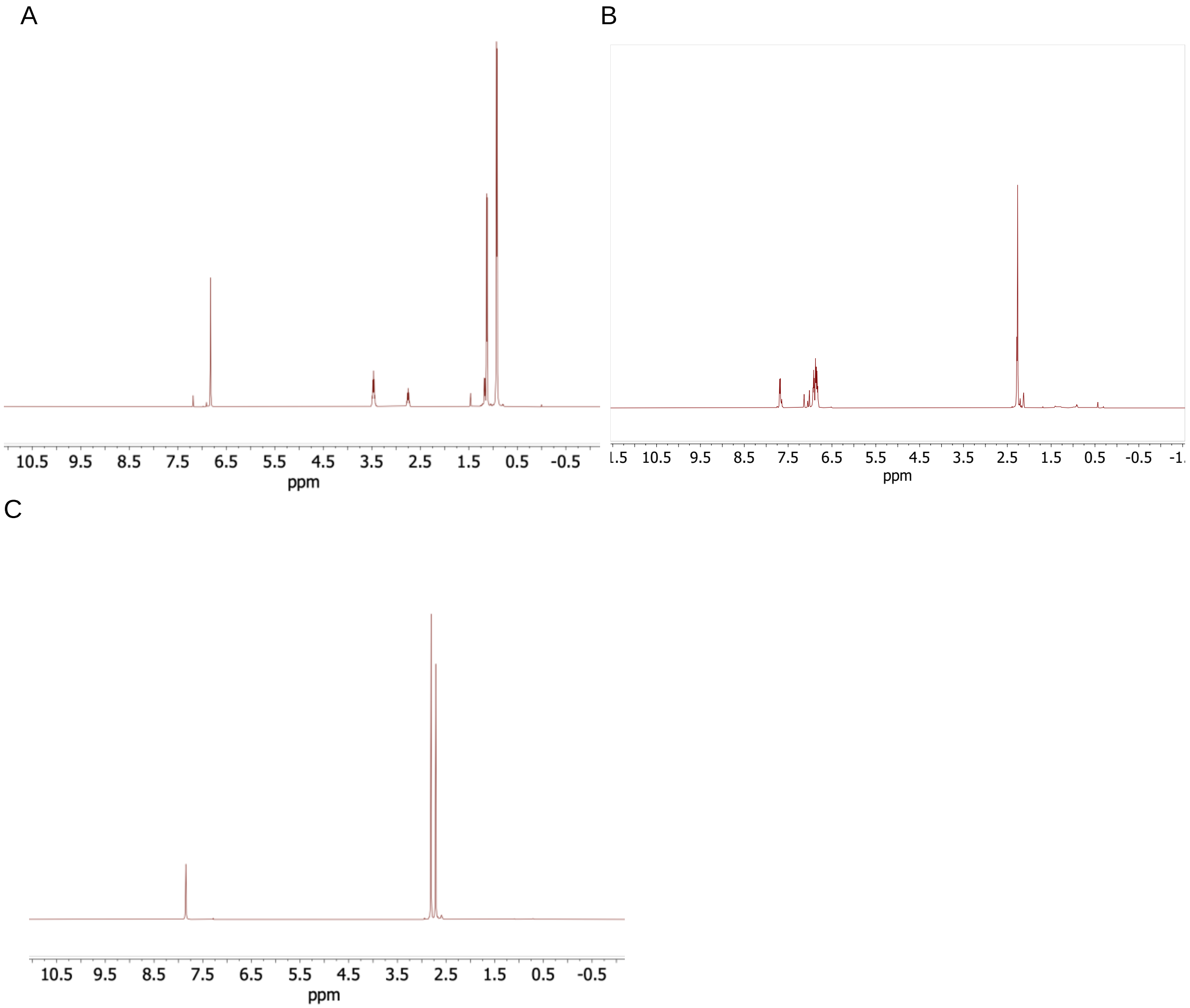

Figure S8. (CDCl$_3$) ^{1}H NMR of A) 1,2-bis(2,4,6-triisopropylphenyl)diselane, B) 1 ,2-di-*o*-tolyldiselane C) 1,4-dihydrobenzo[*d*][1,2]dislenine.

Temperature Dependent Chemical Shifts

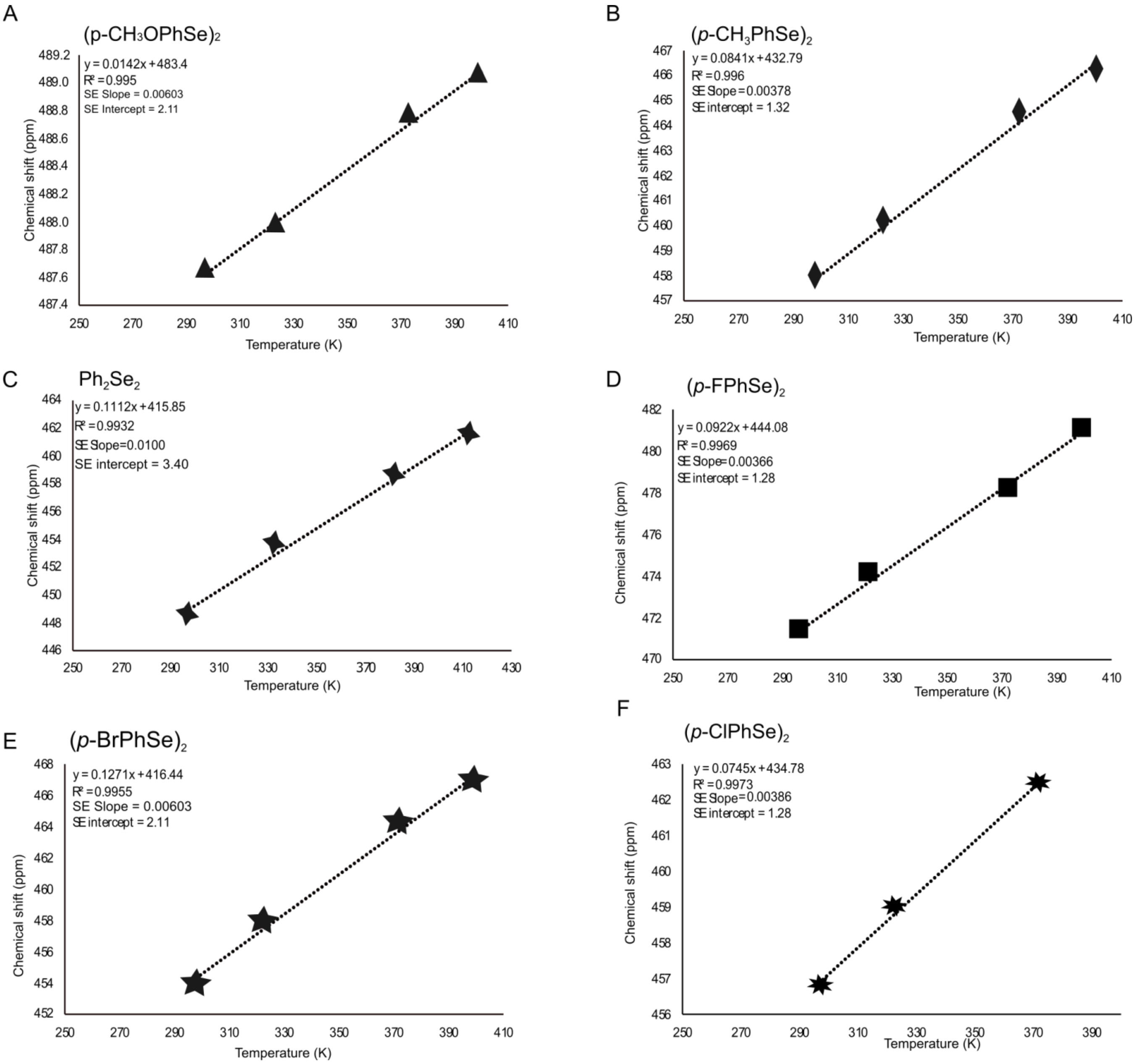

Figure S9. Chemical shift versus temperature in kelvin (K) showing linear trends confirming correlation of data for A) (*p*-$CH_3OPhSe)_2$ B) (*p*-$CH_3PhSe)_2$ C) Ph_2Se_2 D) (*p*-FPhSe$)_2$ E) (*p*-BrPhSe$)_2$ F) (*p*-ClPhSe$)_2$.

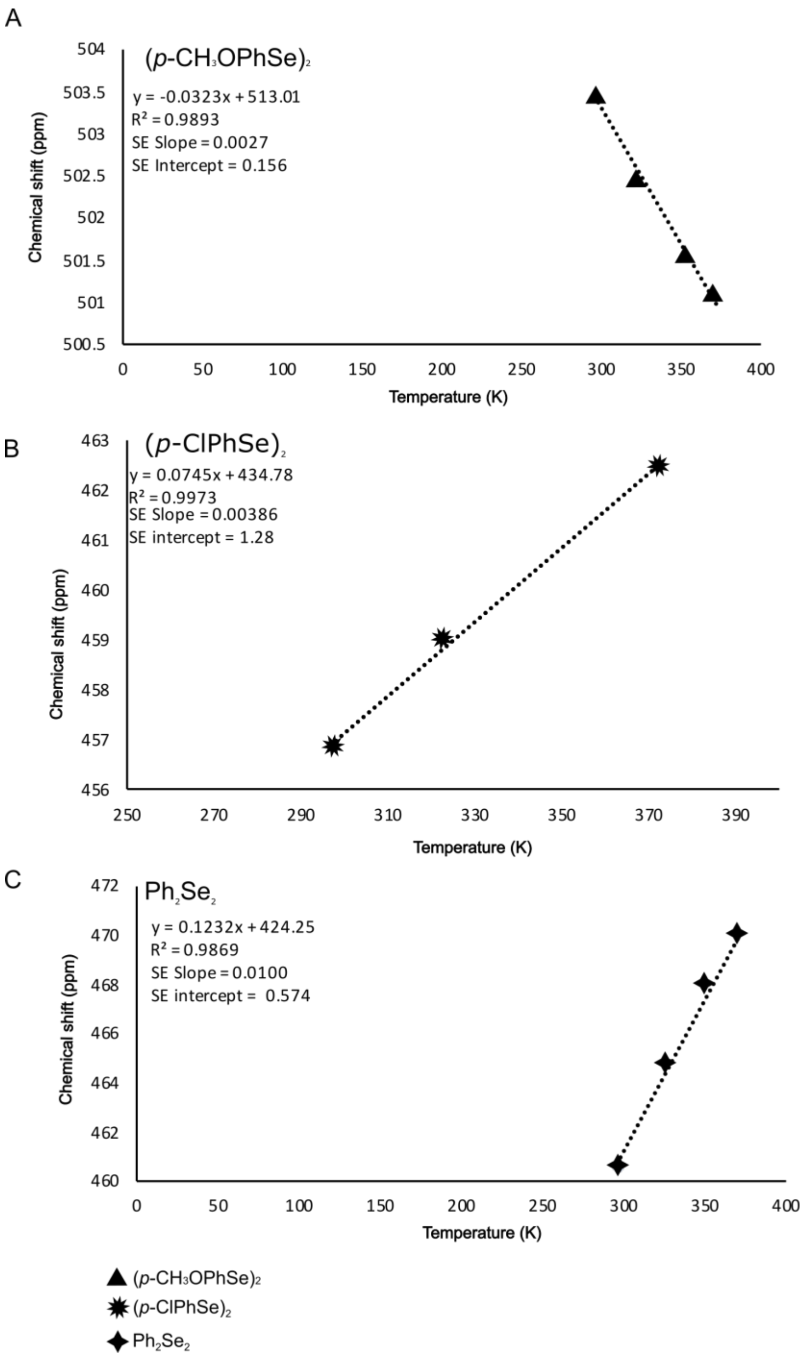

Figure S10. Chemical shift versus temperature in Kelvin (K) showing linear trends confirming correlation of data studied in toluene-d_8 for A) (p-$CH_3OPhSe)_2$ B) (p-$ClPhSe)_2$ C) Ph_2Se_2.

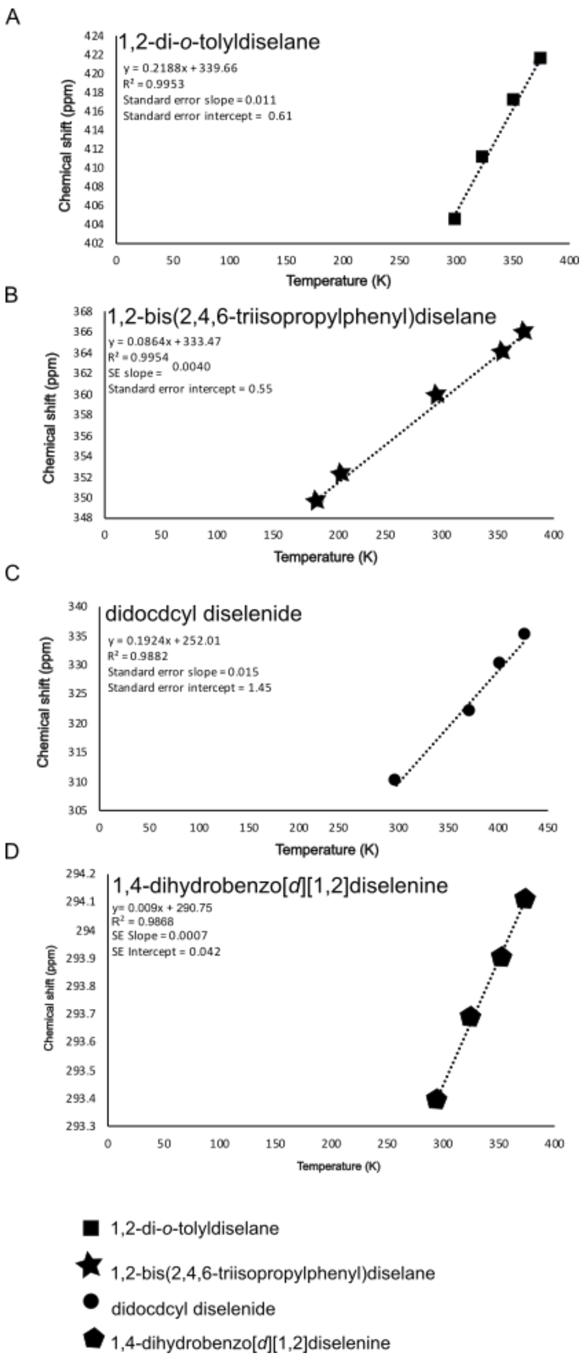

Figure S11. Chemical shift versus temperature in Kelvin (K) showing linear trends confirming correlation of data studied for A) 1,2 di-*o*-tolyldiselane (toluene-d_8) B) 1,2-bis(2,4,6-triisopropylphenyl)diselane (toluene-d_8) C) didodecyl diselenide (deuterated 1,2 dichlorobenzene) D)1,4-dihydrobenzo[*d*][1,2]dislenine (DMSO-d_6).

Dialkyl Diselenide ^{77}Se NMR Spectra

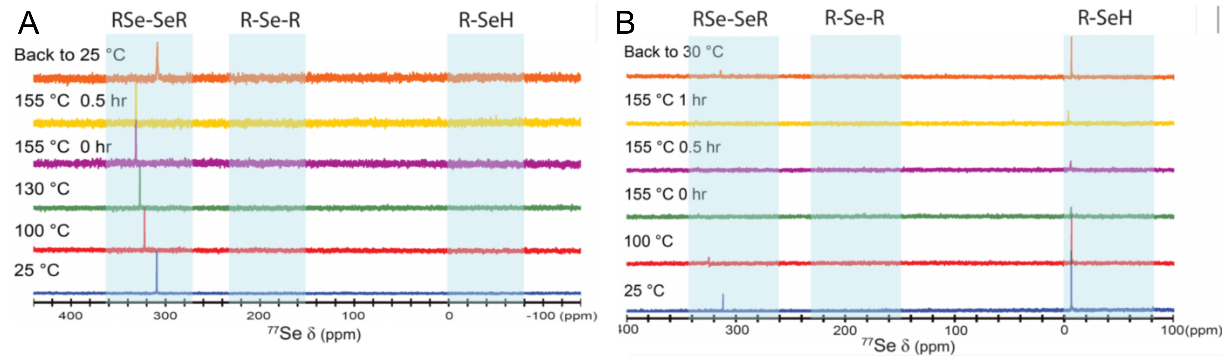

Figure S12. Heated studies were performed at the temperatures and times listed above. A) ^{77}Se NMR of didodecyl diselenide (DD_2Se_2) in deuterated 1,2 dichlorobenzene. B) ^{77}Se NMR of didodecyl diselenol in deuterated 1,2 dichlorobenzene. Didodecyl selenol oxidizes to the diselenide form causing the peak at $\delta = 315$.

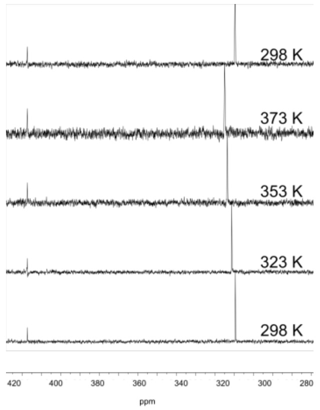

Figure S13. Didodecyl diselenide ^{77}Se NMR heated in deuterated toluene with Ph_2Se internal standard (0.05 mmol).

Coordinates of DFT structures

Table S2. DFT optimized geometries in the gas-phase for differently susbituted diphenyl diselenides. Level of theory B3LYP/cc-pVTZ

Ph2Se2
Total Energy=-5266.73730203 Ha
Nimag=0

Se	-11.549167	1.729937	0.111007
C	-13.385296	1.233017	-0.256669
C	-14.377276	2.208734	-0.348186
C	-15.697246	1.835368	-0.568014
C	-16.032684	0.490729	-0.690717
C	-15.044810	-0.482305	-0.595021
C	-13.721101	-0.114488	-0.379157
H	-14.115480	3.252728	-0.248051
H	-16.464655	2.594824	-0.638815
H	-17.061976	0.203094	-0.858625
H	-15.301879	-1.529104	-0.688990
H	-12.950408	-0.868912	-0.307060
Se	-10.730205	2.296781	-2.042804
C	-10.270231	0.566999	-2.784282
C	-9.037414	-0.019147	-2.499655
C	-8.702109	-1.239190	-3.073358
C	-9.590205	-1.874308	-3.935632
C	-10.817231	-1.288098	-4.223088
C	-11.160184	-0.068872	-3.648680
H	-8.346804	0.477936	-1.832984
H	-7.744877	-1.691596	-2.850336
H	-9.324787	-2.822751	-4.383269
H	-11.510165	-1.778491	-4.893953
H	-12.114810	0.388015	-3.868363

pBr-Ph2Se2
Total Energy=-10413.98184 Ha
Nimag=0

Se	0.68809	2.41879	-0.96579
C	1.96077	1.01339	-0.57381
C	3.04527	1.25156	0.26904
C	3.97766	0.25205	0.51334
C	3.82158	-0.98501	-0.09950
C	2.75185	-1.23873	-0.94599
C	1.81900	-0.23407	-1.17840
H	3.16418	2.21849	0.73722
H	4.81775	0.43561	1.16692
H	2.64295	-2.20504	-1.41600
H	0.97962	-0.42589	-1.83181
Se	-0.68845	2.41905	0.96555
C	-1.96099	1.01341	0.57389
C	-3.04614	1.25180	-0.26803
C	-3.97844	0.25216	-0.51227
C	-3.82142	-0.98526	0.09957
C	-2.75099	-1.23921	0.94512
C	-1.81837	-0.23438	1.17760
H	-3.16565	2.21900	-0.73549
H	-4.81906	0.43584	-1.16511
Br	-5.10592	-2.36078	-0.22674
H	-0.97850	-0.42625	1.83037
H	-2.64149	-2.20583	1.41434
Br	5.10630	-2.36032	0.22686

pCH3-Ph2Se2
Total Energy=-5345.39727 Ha
Nimag=0

Se	0.51950	-1.62111	0.85604
C	1.94538	-0.36784	0.48385
C	3.10611	-0.79251	-0.16077
C	4.14831	0.09858	-0.37589
C	4.06082	1.42820	0.04158
C	2.89597	1.83749	0.69113
C	1.84743	0.95373	0.91411
H	3.19311	-1.81823	-0.49111
H	5.04624	-0.24622	-0.87404
H	2.80742	2.86144	1.03291
H	0.95575	1.28908	1.42484
Se	-0.87037	-1.42448	-1.05873
C	-1.95721	0.12316	-0.63633
C	-1.72073	1.32114	-1.30341
C	-2.53735	2.41789	-1.04378
C	-3.56970	2.32013	-0.12275
C	-3.81627	1.12363	0.55752
C	-3.00148	0.02836	0.28352
H	-0.90882	1.39619	-2.01268
H	-2.36108	3.35214	-1.56072
H	-4.19631	3.18189	0.07241
H	-3.17839	-0.91064	0.79088
C	-4.94217	1.02541	1.55387
H	-4.80854	1.73781	2.37052
H	-5.90358	1.24822	1.08666
H	-5.00346	0.02814	1.98687
C	5.18375	2.39577	-0.22242
H	6.15550	1.90654	-0.15266
H	5.10560	2.81905	-1.22729
H	5.16897	3.22583	0.48330

pCl-Ph2Se2
Total Energy=-6185.99666 Ha
Nimag=0

Se	-0.71999	-1.94051	-0.94303
C	-1.97294	-0.52920	-0.51316
C	-3.04780	-0.76904	0.34187
C	-3.96543	0.23590	0.61493
C	-3.80511	1.48136	0.02009
C	-2.74492	1.73646	-0.83842
C	-1.82736	0.72620	-1.10091
H	-3.16960	-1.74181	0.79686
H	-4.79963	0.05588	1.27717
H	-2.63708	2.71063	-1.29246
H	-0.99622	0.91857	-1.76448
Se	0.72001	-1.94055	0.94299
C	1.97297	-0.52923	0.51320
C	3.04781	-0.76902	-0.34189
C	3.96542	0.23593	-0.61492
C	3.80514	1.48136	-0.01997
C	2.74497	1.73640	0.83857

C 1.82742 0.72613 1.10102
H 3.16959 -1.74178 -0.79693
H 4.79958 0.05598 -1.27723
Cl 4.96226 2.74975 -0.35817
H 0.99631 0.91846 1.76465
H 2.63716 2.71052 1.29276
Cl -4.96237 2.74966 0.35810

pF-Ph2Se2
Total Energy=-5465.28428 Ha
Nimag=0
Se -0.75942 -1.64557 -0.91311
C -1.99198 -0.23187 -0.44061
C -3.06113 -0.48122 0.42031
C -3.96744 0.52541 0.72429
C -3.79012 1.77364 0.15092
C -2.74291 2.04912 -0.71049
C -1.83907 1.03502 -1.00366
H -3.18521 -1.46306 0.85440
H -4.80239 0.35420 1.38870
H -2.64371 3.03710 -1.13698
H -1.01286 1.23041 -1.67222
Se 0.75935 -1.64550 0.91316
C 1.99193 -0.23184 0.44059
C 3.06103 -0.48127 -0.42037
C 3.96741 0.52529 -0.72437
C 3.79020 1.77352 -0.15097
C 2.74307 2.04907 0.71049
C 1.83916 1.03505 1.00369
H 3.18501 -1.46311 -0.85448
H 4.80234 0.35404 -1.38880
F 4.66922 2.75211 -0.44085
H 1.01298 1.23050 1.67227
H 2.64395 3.03706 1.13698
F -4.66904 2.75230 0.44080

pOCH3-Ph2Se2
Total Energy=-5495.86834 Ha
Nimag=0
Se -0.74117 -1.96675 -0.89194
C -1.95484 -0.50422 -0.55581
C -3.08277 -0.68359 0.23821
C -3.97971 0.35769 0.45210
C -3.75014 1.59994 -0.14088
C -2.62086 1.78452 -0.94400
C -1.73366 0.74343 -1.14712
H -3.26679 -1.64413 0.69899
H -4.84565 0.18908 1.07385
H -2.46030 2.75271 -1.39772
H -0.86225 0.89526 -1.76841
Se 0.71529 -1.90480 0.99149
C 2.02783 -0.58147 0.49192
C 3.12338 -0.92293 -0.30773
C 4.08226 0.01971 -0.62412
C 3.97004 1.32832 -0.14155
C 2.88374 1.67722 0.66010
C 1.92038 0.72069 0.96844
H 3.22032 -1.93238 -0.68216
H 4.93293 -0.23310 -1.24170
H 1.07827 0.99772 1.58710
O -4.55981 2.68203 0.00015
C -5.72642 2.56243 0.79995
H -6.21052 3.53463 0.76886
H -6.40846 1.80692 0.40198
H -5.47778 2.31582 1.83519
H 2.77475 2.67924 1.04607
O 4.96418 2.17965 -0.50569
C 4.91118 3.52377 -0.05312
H 4.01472 4.03258 -0.41639
H 4.94169 3.58008 1.03793
H 5.79154 4.01156 -0.46235

Table S3 DFT constrained optimized geometries in the gas-phase, toluene, and DMSO for Ph_2Se_2 and $pOCH_3$-Ph_2Se_2 with different values of Ψ dihedral. Level of theory (SMD)-B3LYP/cc-pVTZ

Ph2Se2_Psi_0
Total Energy=-5266.72294 Ha
Nimag=0
Se -1.10285 -1.86261 0.00012
C -1.68838 -0.02963 0.00016
C -1.94425 0.62128 1.20764
C -2.45838 1.91218 1.20429
C -2.71667 2.55793 0.00025
C -2.45943 1.91185 -1.20383
C -1.94528 0.62096 -1.20728
H -1.74882 0.11272 2.14080
H -2.65948 2.41163 2.14270
H -2.66134 2.41104 -2.14222
H -1.75063 0.11215 -2.14047
Se 1.35664 -1.68812 -0.00012
C 1.67661 0.20930 -0.00016
C 1.83700 0.89004 -1.20764
C 2.16103 2.24127 -1.20428
C 2.32396 2.91738 -0.00025
C 2.16212 2.24109 1.20382
C 1.83807 0.88986 1.20728
H 1.71636 0.35875 -2.14080
H 2.28860 2.76436 -2.14269
H 1.71825 0.35845 2.14047
H 2.29054 2.76403 2.14220
H 2.57877 3.96878 -0.00029
H -3.11956 3.56195 0.00029

Ph2Se2_Psi_180
Total Energy=-5266.72682 Ha
Nimag=0
Se -0.55551 0.01131 -1.08145
C -2.36746 0.00508 -0.41155
C -3.02942 1.21053 -0.17919
C -4.35000 1.20325 0.25422
C -5.01169 -0.00274 0.45740
C -4.35158 -1.20483 0.22776
C -3.03103 -1.20432 -0.20585
H -2.51126 2.14537 -0.33903

```
H  -4.86105     2.14014     0.43187
H  -6.03991    -0.00575     0.79371
H  -4.86383    -2.14472     0.38482
H  -2.51419    -2.13611    -0.38643
Se   0.55550    -0.01555     1.08136
C   2.36745    -0.00489     0.41152
C   3.03283    -1.20875     0.18074
C   4.35345    -1.19828    -0.25256
C   5.01171     0.00930    -0.45724
C   4.34816     1.20982    -0.22918
C   3.02763     1.20613     0.20435
H   2.51729    -2.14485     0.34167
H   4.86713    -2.13395    -0.42901
H   6.03994     0.01483    -0.79348
H   4.85780     2.15096    -0.38738
H   2.50815     2.13667     0.38375
```

```
Ph2Se2_Psi_180_DMSO
Total Energy=-5266.73672 Ha
Nimag=0
Se  -0.56264    -0.01993    -1.07831
C  -2.37536    -0.00658    -0.40879
C  -3.03347     1.20687    -0.20777
C  -4.35495     1.21194     0.22527
C  -5.01872     0.01155     0.45842
C  -4.36046    -1.19788     0.25887
C  -3.03892    -1.21093    -0.17391
H  -2.51776     2.13920    -0.39114
H  -4.86474     2.15408     0.37838

H  -6.04737     0.01858     0.79387
H  -4.87447    -2.13305     0.43839
H  -2.52736    -2.15033    -0.33091
Se   0.56252     0.01550     1.07803
C   2.37542     0.00659     0.40898
C   3.03646    -1.20510     0.20713
C   4.35803    -1.20668    -0.22576
C   5.01885    -0.00454    -0.45819
C   4.35764     1.20318    -0.25783
C   3.03611     1.21273     0.17496
H   2.52299    -2.13881     0.38977
H   4.87001    -2.14750    -0.37961
H   6.04751    -0.00883    -0.79364
H   4.86945     2.13970    -0.43660
H   2.52232     2.15079     0.33268
```

```
Ph2Se2_Psi_180_toluene
Total Energy=-5266.74032 Ha
Nimag=0
Se  -0.56053    -0.06914    -1.07711
C  -2.37324    -0.02487    -0.40842
C  -3.02886     1.19639    -0.25327
C  -4.34976     1.22184     0.17893
C  -5.01703     0.03359     0.45647
C  -4.36243    -1.18376     0.30295
C  -3.04129    -1.21673    -0.12824
H  -2.50785     2.11801    -0.47142
H  -4.85663     2.17047     0.29714
H  -6.04557     0.05627     0.79145
H  -4.87922    -2.10984     0.51773
H  -2.52977    -2.16125    -0.24913
Se   0.56049     0.06493     1.07722
C   2.37323     0.02509     0.40838
C   3.03179    -1.19460     0.25337
C   4.35273    -1.21693    -0.17887
C   5.01712    -0.02711    -0.45659
C   4.35958     1.18868    -0.30324
C   3.03838     1.21853     0.12800
H   2.51300    -2.11744     0.47168
H   4.86188    -2.16434    -0.29698
H   6.04571    -0.04736    -0.79159
H   4.87415     2.11597    -0.51814
H   2.52459     2.16183     0.24875
```

```
pOCH3-Ph2Se2_Psi_180
Total Energy=-5495.86126 Ha
Nimag=0
Se  -0.48070     0.16741    -1.11652
C  -2.33373     0.14807    -0.60070
C  -3.00443     1.34120    -0.31019
C  -4.34809     1.33116     0.01170
C  -5.05363     0.12392     0.05064
C  -4.39343    -1.07131    -0.23573
C  -3.04004    -1.05015    -0.55704
H  -2.46582     2.27777    -0.33967
H  -4.87627     2.24733     0.23630
H  -4.91517    -2.01574    -0.21230
H  -2.53145    -1.97823    -0.77691
Se   0.47880    -0.01062     1.12089
C   2.33247     0.05300     0.61066
C   3.04685    -1.12480     0.41348
C   4.40142    -1.09520     0.09744
C   5.05395     0.13140    -0.02874
C   4.34002     1.31875     0.16494
C   2.99576     1.27819     0.48164
H   2.54375    -2.07682     0.50759
H   4.92970    -2.02510    -0.04715
H   4.86262     2.25972     0.06375
H   2.45104     2.19937     0.63270
O  -6.36897     0.21801     0.37459
C  -7.14427    -0.96974     0.43422
H  -7.17492    -1.47578    -0.53392
H  -6.76160    -1.65894     1.19105
H  -8.14817    -0.65726     0.70791
O   6.36932     0.27564    -0.33360
C   7.15259    -0.88895    -0.54703
H   7.18531    -1.51774     0.34621
H   6.77558    -1.47508    -1.38878
H   8.15478    -0.53677    -0.77498
```

```
pOCH3-Ph2Se2_Psi_180_DMSO
Total Energy=-5495.87469 Ha
Nimag=0
Se  -0.49894     0.13029    -1.11733
C  -2.35024     0.13157    -0.59838
C  -3.00625     1.33361    -0.30989
C  -4.34968     1.33698     0.01524
C  -5.06937     0.13569     0.05787
C  -4.42073    -1.06811    -0.22845
C  -3.06798    -1.06117    -0.55105
H  -2.46282     2.26777    -0.34260
H  -4.86231     2.26323     0.23778
```

```
H  -4.95337    -2.00646    -0.20376
H  -2.57384    -1.99781    -0.76964
Se   0.49645     0.00637     1.11524
C   2.34866     0.06605     0.60212
C   3.07133    -1.11448     0.44700
C   4.42540    -1.08635     0.13067
C   5.07004     0.14132    -0.04002
C   4.34503     1.33065     0.11122
C   3.00061     1.29218     0.42979
H   2.58027    -2.06926     0.57527
H   4.96160    -2.01654     0.02036
H   4.85452     2.27562    -0.02147
H   2.45385     2.21738     0.54854
O  -6.37817     0.23963     0.38265
C  -7.16484    -0.95252     0.44638
H  -7.20090    -1.45600    -0.52163
H  -6.78197    -1.63976     1.20330
H  -8.16581    -0.63227     0.72348
O   6.37964     0.28060    -0.34830
C   7.17458    -0.89661    -0.51019
H   7.20078    -1.48656     0.40793
H   6.80642    -1.51349    -1.33214
H   8.17710    -0.54728    -0.74288
```

pOCH3-Ph2Se2_Psi_180_DMSO_anti
Total Energy=-5495.87471 Ha
Nimag=0

```
Se  -0.49335    -0.42522    -1.03572
C  -2.34417    -0.33426    -0.52248
C  -3.09585     0.80668    -0.79358
C  -4.44821     0.86347    -0.47466
C  -5.06311    -0.23669     0.12859
C  -4.30946    -1.38457     0.40582
C  -2.96586    -1.43131     0.08418
H  -2.62894     1.66282    -1.26077
H  -5.00586     1.75972    -0.69958
H  -4.79574    -2.23073     0.87242
H  -2.39615    -2.32410     0.30191
Se   0.49375     0.40998     1.04206
C   2.34381     0.33043     0.52422
C   3.10236    -0.80658     0.79270
C   4.45428    -0.85571     0.47062
C   5.06172     0.24824    -0.13320
C   4.30113     1.39222    -0.40774
C   2.95805     1.43137    -0.08296
H   2.64122    -1.66562     1.26025
H   5.01735    -1.74906     0.69355
H   4.78175     2.24142    -0.87470
H   2.38298     2.32123    -0.29863
O  -6.36884    -0.28634     0.47755
C  -7.19172     0.85388     0.21920
H  -7.24031     1.07355    -0.84899
H  -8.18330     0.59084     0.57820
H  -6.83255     1.73235     0.75857
O   6.36634     0.30528    -0.48519
C   7.19585    -0.83081    -0.22982
H   7.24820    -1.05112     0.83806
H   6.83996    -1.71069    -0.76909
H   8.18514    -0.56226    -0.59104
```

pOCH3-Ph2Se2_Psi_180_anti
Total Energy=-5495.86127 Ha
Nimag=0

```
Se   0.47721     0.42382    -1.04190
C   2.32915     0.33244    -0.52987
C   3.07891    -0.80948    -0.79564
C   4.43147    -0.86750    -0.47583
C   5.04717     0.23243     0.12228
C   4.29811     1.38193     0.39442
C   2.95524     1.42936     0.07208
H   2.60509    -1.66382    -1.25815
H   4.98728    -1.76598    -0.69604
H   4.79211     2.22439     0.85810
H   2.38268     2.32105     0.28467
Se  -0.47705    -0.40078     1.04821
C  -2.32815    -0.32578     0.53047
C  -3.09068     0.80579     0.80376
C  -4.44304     0.85201     0.48088
C  -5.04521    -0.24910    -0.12856
C  -4.28308    -1.38817    -0.40861
C  -2.94084    -1.42422    -0.08250
H  -2.62721     1.66127     1.27457
H  -5.00905     1.74257     0.70720
H  -4.76677    -2.23180    -0.88095
H  -2.35826    -2.30804    -0.30065
O   6.35770     0.28356     0.47423
C   7.17532    -0.85095     0.22959
H   7.22692    -1.08366    -0.83693
H   8.16651    -0.58715     0.58780
H   6.81579    -1.72734     0.77446
O  -6.35408    -0.31122    -0.48481
C  -7.18475     0.81197    -0.23203
H  -7.24148     1.03451     0.83640
H  -6.83361     1.69713    -0.76811
H  -8.17211     0.54049    -0.59508
```

pOCH3-Ph2Se2_Psi_180_toluene
Total Energy=-5495.87576 Ha
Nimag=0

```
Se  -0.49232     0.14771    -1.12493
C  -2.34444     0.13816    -0.60524
C  -3.01149     1.33605    -0.32608
C  -4.35410     1.33103     0.00079
C  -5.06195     0.12473     0.05581
C  -4.40398    -1.07479    -0.22124
C  -3.05194    -1.05927    -0.54676
H  -2.47320     2.27261    -0.36722
H  -4.87748     2.25229     0.21735
H  -4.92824    -2.01751    -0.18729
H  -2.54697    -1.99154    -0.75819
Se   0.48645     0.01366     1.11083
C   2.34087     0.06625     0.60206
C   3.05306    -1.11634     0.42306
C   4.40842    -1.09389     0.11167
C   5.06493     0.13001    -0.02854
C   4.35167     1.32175     0.14586
C   3.00602     1.28875     0.45807
H   2.54935    -2.06708     0.52861
H   4.93535    -2.02675    -0.01864
H   4.87385     2.26220     0.03465
H   2.46442     2.21433     0.59433
O  -6.37294     0.22174     0.38392
```

```
C  -7.14778    -0.96969     0.47062
H  -7.19112    -1.48964    -0.48939
H  -6.75777    -1.64531     1.23559
H  -8.14947    -0.65335     0.75063
O   6.37924     0.26543    -0.32872
C   7.16564    -0.90754    -0.51008
H   7.18695    -1.51845     0.39548
H   6.80209    -1.50720    -1.34785
H   8.17189    -0.55999    -0.73017
```

```
pOCH3-Ph2Se2_Psi_180_toluene_anti
Total Energy=-5495.87578 Ha
Nimag=0
Se  -0.48762    -0.42671    -1.03579
C  -2.33905    -0.33376    -0.52187
C  -3.08923     0.80721    -0.79273
C  -4.44191     0.86485    -0.47487
C  -5.05807    -0.23413     0.12642
C  -4.30695    -1.38208     0.40406
C  -2.96352    -1.42957     0.08381
H  -2.61758     1.66140    -1.25822
H  -4.99843     1.76201    -0.69900
H  -4.79797    -2.22539     0.87015
H  -2.39181    -2.32095     0.30110
Se   0.48806     0.41035     1.04282
C   2.33866     0.32955     0.52382
C   3.09718    -0.80566     0.79555
C   4.44937    -0.85510     0.47408
C   5.05640     0.24627    -0.13203
C   4.29684     1.38851    -0.41045
C   2.95410     1.42799    -0.08635
H   2.63252    -1.66178     1.26451
H   5.01249    -1.74790     0.69908
H   4.78095     2.23384    -0.88009
H   2.37590     2.31503    -0.30417
O  -6.36643    -0.28448     0.47485
C  -7.18901     0.84906     0.21825
H  -7.23970     1.07244    -0.85001
H  -8.18108     0.58332     0.57463
H  -6.83708     1.72977     0.76055
O   6.36333     0.30437    -0.48463
C   7.19400    -0.82334    -0.22828
H   7.24995    -1.04384     0.84030
H   6.84568    -1.70752    -0.76726
H   8.18307    -0.55221    -0.58889
```

Polarity analysis:

The calculations showed that for Ph_2Se_2 the dipole difference from Ψ=90° to Ψ =180° is of more than 2D. The magnitude of the difference increases as the polarity of the environment increases. The structures with Ψ =90° are the true minima of the systems and thus are energetically favored. For ($p$$OCH_3$)$_2$-$Ph_2Se_2$, the relative energy in the Ψ =90° structures in the *syn* and *anti* conformation is determined by the solvent: in the gas phase and in toluene, two apolar media, the syn and anti conformer are found at the same energy, despite having different dipoles (the structures with the lower dipoles are actually more favored, albeit only by a few hundredths of kcal mol^{-1}). In DMSO, a more polar environment, we see a slight stabilization of the structure with the highest dipole (0.24 kcal mol^{-1}).

For ($p$$OCH_3$)$_2$-$Ph_2Se_2$ the dipole difference from Ψ =90° to Ψ =180° is larger in the *anti* conformation and again increases with the polarity of the medium. Despite a noticeable difference in dipole moments for the syn and *anti* conformers the differences in electronic energies are negligible. A deeper discussion on the energies differences of the syn and *anti* conformers of the Ψ =180° structures is however not useful as these were obtained with a constrained optimization setting the Ψ dihedral at the arbitrary values of 180° for all solvents.

Comparing Ph_2Se_2 and ($p$$OCH_3$)$_2$-$Ph_2Se_2$ (*anti*) we can see that the energy difference to go from 90° to 180° (E_{180}-E_{90}) is of 4.76, 4.51, and 4.15 kcal mol^{-1} for Ph_2Se_2 and of 4.44, 4.32, and 3.86 kcal mol^{-1} for ($p$$OCH_3$)$_2$-$Ph_2Se_2$ in the gas-phase, toluene and DMSO respectively. This means that is easier for ($p$$OCH_3$)$_2$-$Ph_2Se_2$ to adopt the Ψ =180° than Ph_2Se_2 and that this effect is magnified in more polar solvents. Again, this should be treated as a qualitative analysis confirming the effect of the solvent polarity on the and rigorous energy values should be obtained using the true transition state structures.

References:

(1) Eggert, H.; Nielsen, O.; Henriksen, L. ^{77}Se NMR. Application of Se-Se to the Analysis of Dialkyl Polyselenides. *J Am Chem Soc* **1986**, *108* (8), 1725–1730. https://doi.org/10.1021/ja00268a001.
